# Supplementary figures and images for: Ait1 regulates TORC1 signaling and localization in budding yeast
Source: eLife. 2022 Sep 1;11:e68773. doi: 10.7554/eLife.68773 (PMC9499541; doi:10.7554/eLife.68773)

Kog1-FLAG IPs +/- nutrients

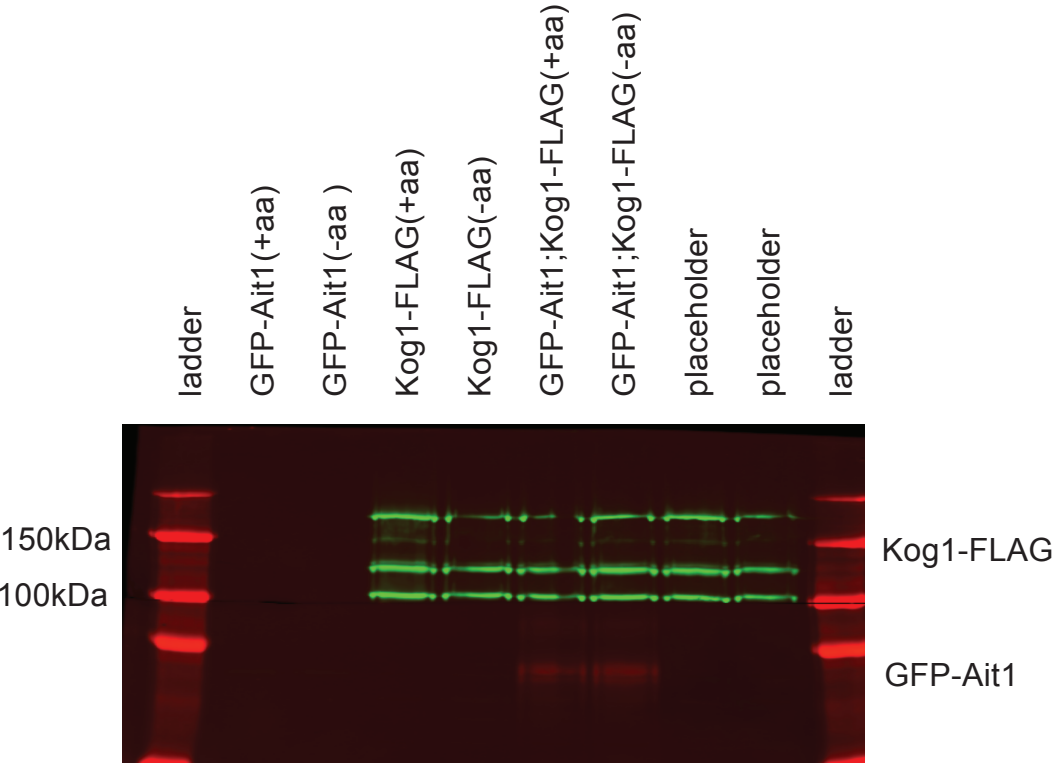

Supplement: Source data 1. — The original labeled gel, from each panel in Figure 3—figure supplement 1, Figure 6, Figure 6—figure supplement 1, Figure 7, Figure 8, Figure 8—figure supplement 3, Figure 9, and Figure 9—figure supplement 1 are included in source data in two separate folders. In each case the gels are numbered as they are shown in the associated figure—from top to bottom. In the case of Figure 6, the two gels on the left are labeled 1 and 2 and the two gels on the right are labeled 3 and 4. [file elife-68773-data1.zip › labelled gel figures/Figure3-figure supplement 1-source data 1 label.pdf]

## Ait1C4 mutant

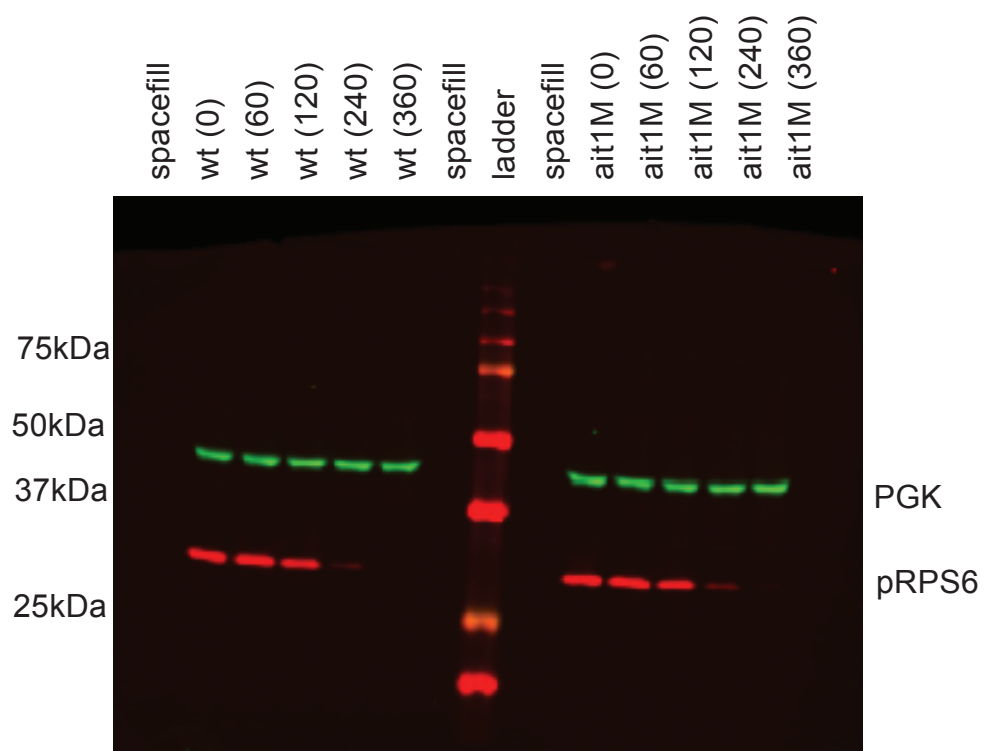

Supplement: Source data 1. — The original labeled gel, from each panel in Figure 3—figure supplement 1, Figure 6, Figure 6—figure supplement 1, Figure 7, Figure 8, Figure 8—figure supplement 3, Figure 9, and Figure 9—figure supplement 1 are included in source data in two separate folders. In each case the gels are numbered as they are shown in the associated figure—from top to bottom. In the case of Figure 6, the two gels on the left are labeled 1 and 2 and the two gels on the right are labeled 3 and 4. [file elife-68773-data1.zip › labelled gel figures/Figure8-source data 1 label.pdf]

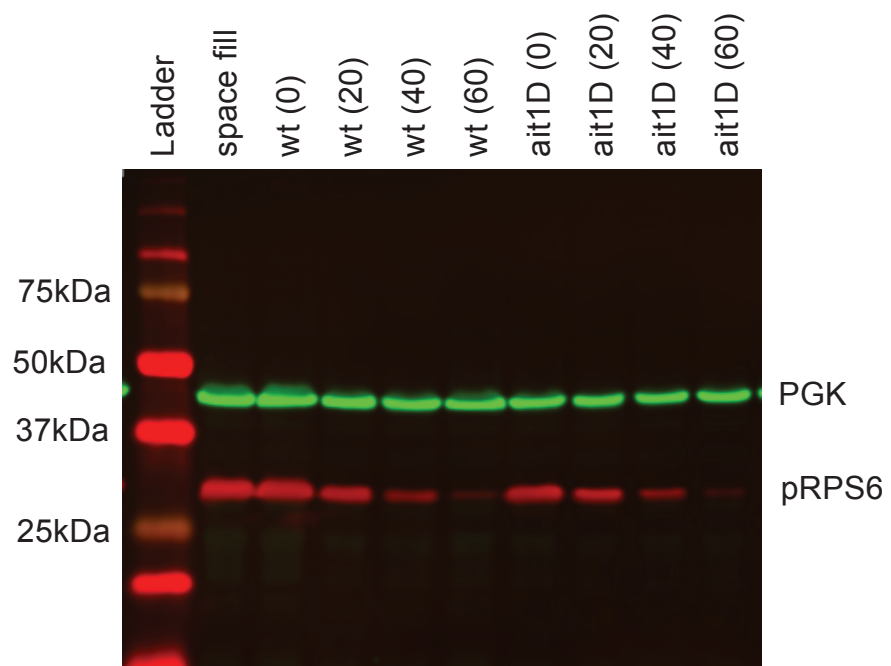

Supplement: Source data 1. — The original labeled gel, from each panel in Figure 3—figure supplement 1, Figure 6, Figure 6—figure supplement 1, Figure 7, Figure 8, Figure 8—figure supplement 3, Figure 9, and Figure 9—figure supplement 1 are included in source data in two separate folders. In each case the gels are numbered as they are shown in the associated figure—from top to bottom. In the case of Figure 6, the two gels on the left are labeled 1 and 2 and the two gels on the right are labeled 3 and 4. [file elife-68773-data1.zip › labelled gel figures/Figure6-figure supplement 1-source data 1 label.pdf]

# Gtr1 off background

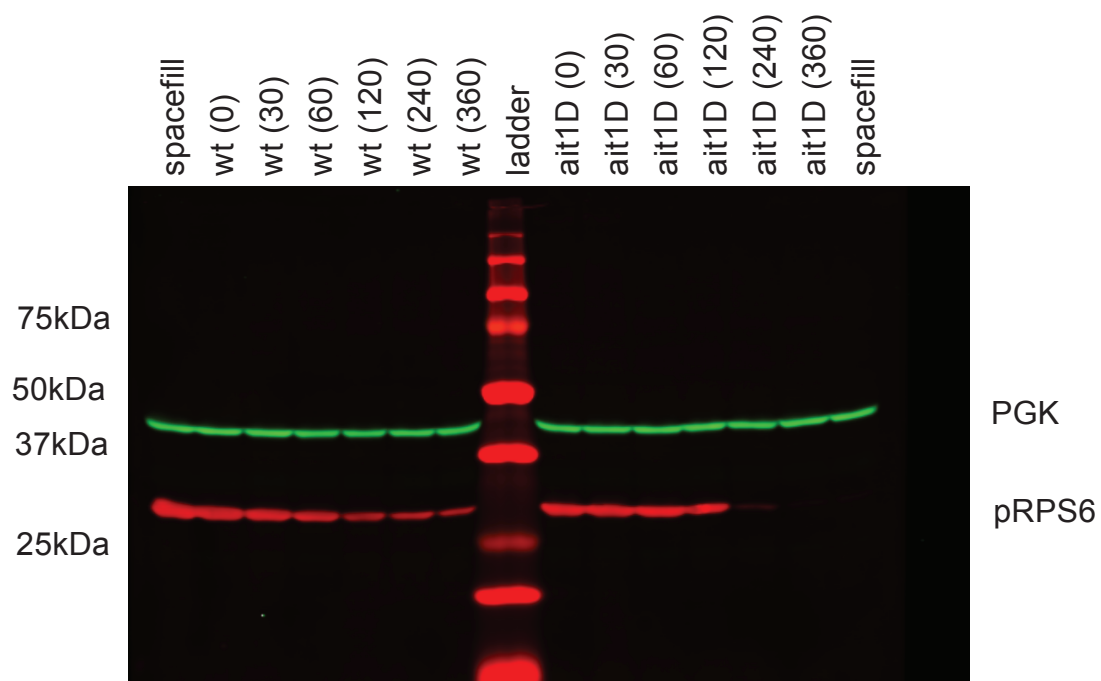

Supplement: Source data 1. — The original labeled gel, from each panel in Figure 3—figure supplement 1, Figure 6, Figure 6—figure supplement 1, Figure 7, Figure 8, Figure 8—figure supplement 3, Figure 9, and Figure 9—figure supplement 1 are included in source data in two separate folders. In each case the gels are numbered as they are shown in the associated figure—from top to bottom. In the case of Figure 6, the two gels on the left are labeled 1 and 2 and the two gels on the right are labeled 3 and 4. [file elife-68773-data1.zip › labelled gel figures/Figure7-source data 1 label.pdf]

Kog1-FLAG IPs +/- nutrients

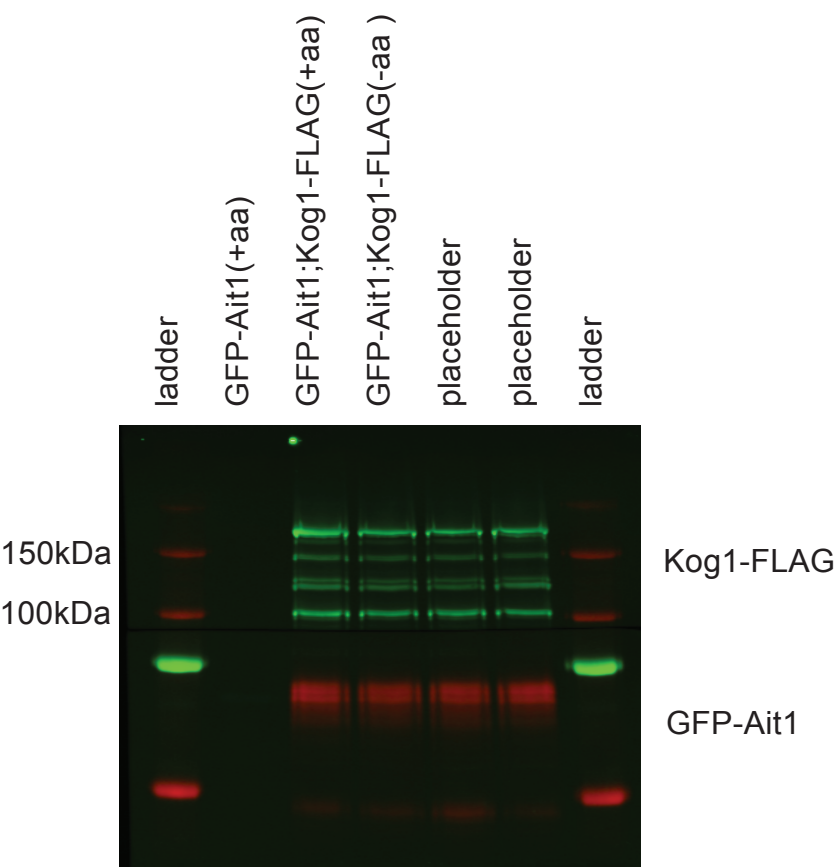

Supplement: Source data 1. — The original labeled gel, from each panel in Figure 3—figure supplement 1, Figure 6, Figure 6—figure supplement 1, Figure 7, Figure 8, Figure 8—figure supplement 3, Figure 9, and Figure 9—figure supplement 1 are included in source data in two separate folders. In each case the gels are numbered as they are shown in the associated figure—from top to bottom. In the case of Figure 6, the two gels on the left are labeled 1 and 2 and the two gels on the right are labeled 3 and 4. [file elife-68773-data1.zip › labelled gel figures/Figure3-figure supplement 1-source data 7 label.pdf]

amino acid starvation

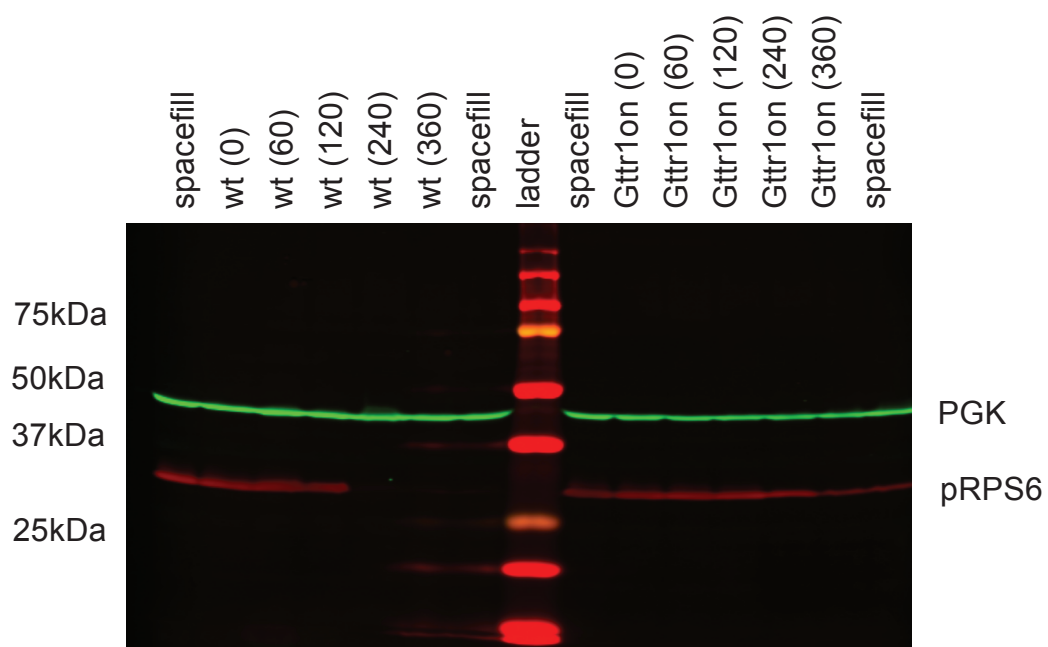

Supplement: Source data 1. — The original labeled gel, from each panel in Figure 3—figure supplement 1, Figure 6, Figure 6—figure supplement 1, Figure 7, Figure 8, Figure 8—figure supplement 3, Figure 9, and Figure 9—figure supplement 1 are included in source data in two separate folders. In each case the gels are numbered as they are shown in the associated figure—from top to bottom. In the case of Figure 6, the two gels on the left are labeled 1 and 2 and the two gels on the right are labeled 3 and 4. [file elife-68773-data1.zip › labelled gel figures/Figure6-source data 3 label.pdf]

## Prm1+2 mutant background

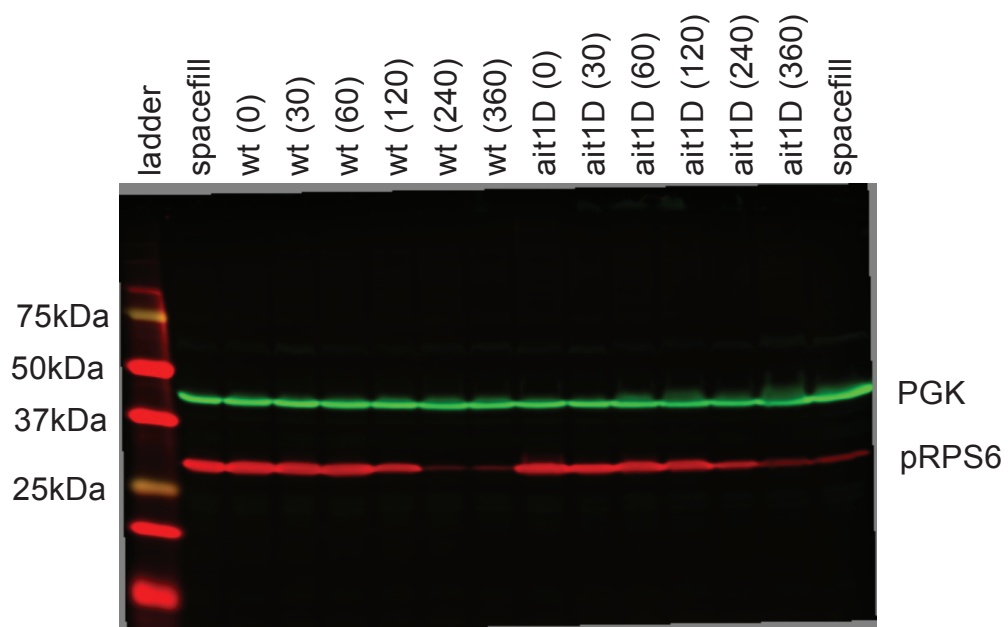

Supplement: Source data 1. — The original labeled gel, from each panel in Figure 3—figure supplement 1, Figure 6, Figure 6—figure supplement 1, Figure 7, Figure 8, Figure 8—figure supplement 3, Figure 9, and Figure 9—figure supplement 1 are included in source data in two separate folders. In each case the gels are numbered as they are shown in the associated figure—from top to bottom. In the case of Figure 6, the two gels on the left are labeled 1 and 2 and the two gels on the right are labeled 3 and 4. [file elife-68773-data1.zip › labelled gel figures/Figure7-source data 7 label.pdf]

# Gtr1-Myc IPs +/- amino acids

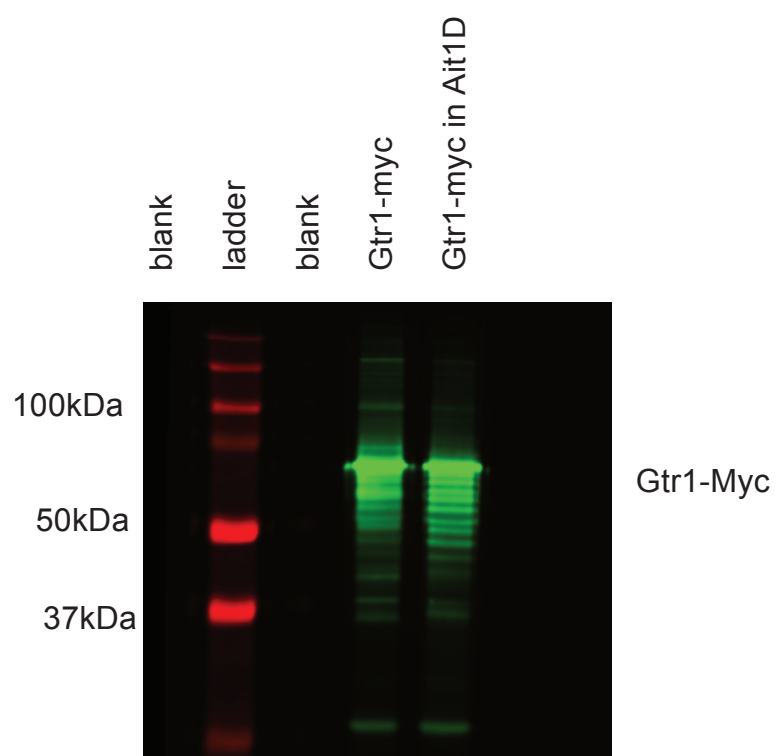

Supplement: Source data 1. — The original labeled gel, from each panel in Figure 3—figure supplement 1, Figure 6, Figure 6—figure supplement 1, Figure 7, Figure 8, Figure 8—figure supplement 3, Figure 9, and Figure 9—figure supplement 1 are included in source data in two separate folders. In each case the gels are numbered as they are shown in the associated figure—from top to bottom. In the case of Figure 6, the two gels on the left are labeled 1 and 2 and the two gels on the right are labeled 3 and 4. [file elife-68773-data1.zip › labelled gel figures/Figure9-figure supplement 1-source data 1 label.pdf]

leucine starvation

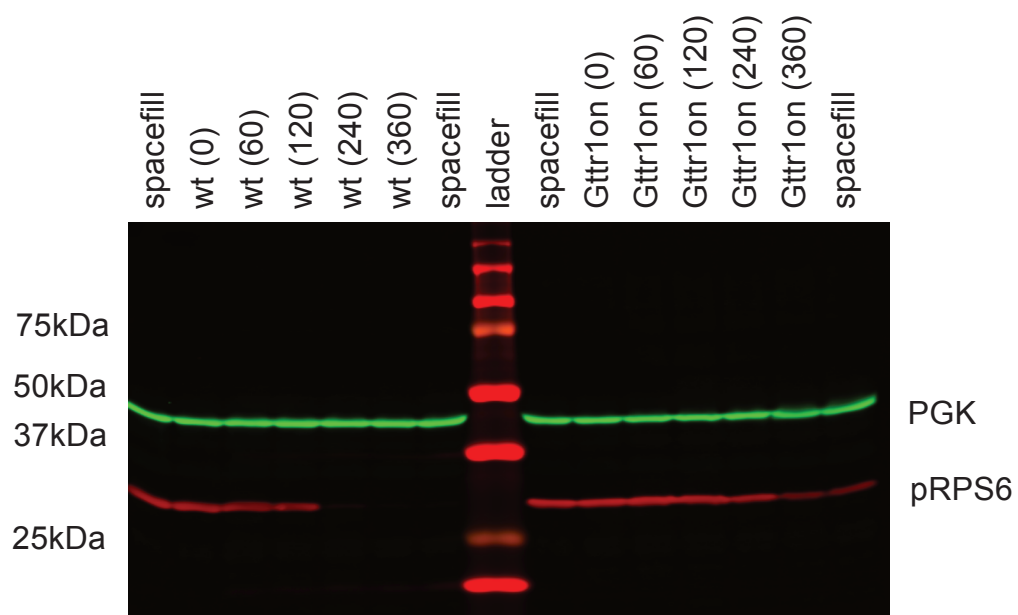

Supplement: Source data 1. — The original labeled gel, from each panel in Figure 3—figure supplement 1, Figure 6, Figure 6—figure supplement 1, Figure 7, Figure 8, Figure 8—figure supplement 3, Figure 9, and Figure 9—figure supplement 1 are included in source data in two separate folders. In each case the gels are numbered as they are shown in the associated figure—from top to bottom. In the case of Figure 6, the two gels on the left are labeled 1 and 2 and the two gels on the right are labeled 3 and 4. [file elife-68773-data1.zip › labelled gel figures/Figure6-source data 4 label.pdf]

## Ait1v2 mutant replicate

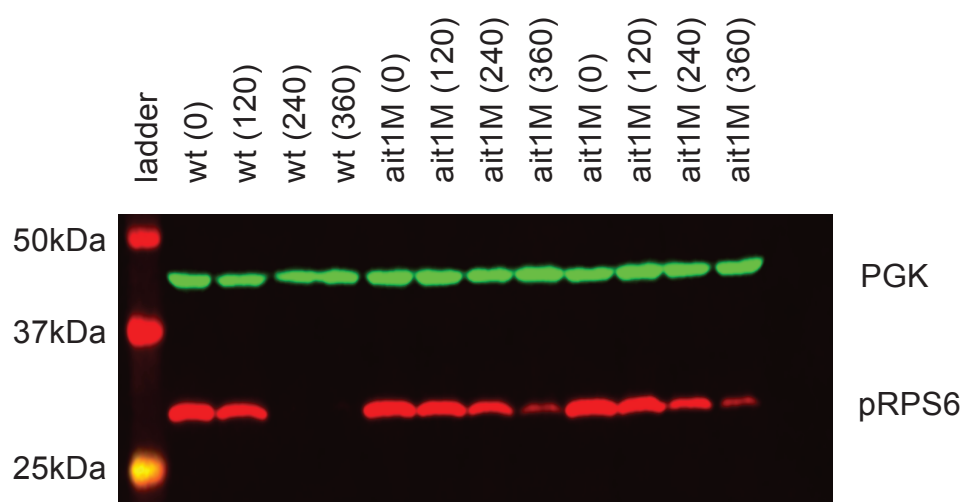

Supplement: Source data 1. — The original labeled gel, from each panel in Figure 3—figure supplement 1, Figure 6, Figure 6—figure supplement 1, Figure 7, Figure 8, Figure 8—figure supplement 3, Figure 9, and Figure 9—figure supplement 1 are included in source data in two separate folders. In each case the gels are numbered as they are shown in the associated figure—from top to bottom. In the case of Figure 6, the two gels on the left are labeled 1 and 2 and the two gels on the right are labeled 3 and 4. [file elife-68773-data1.zip › labelled gel figures/Figure8-figure supplement 3-source data 1 label.pdf]

PGK +/- nutrients

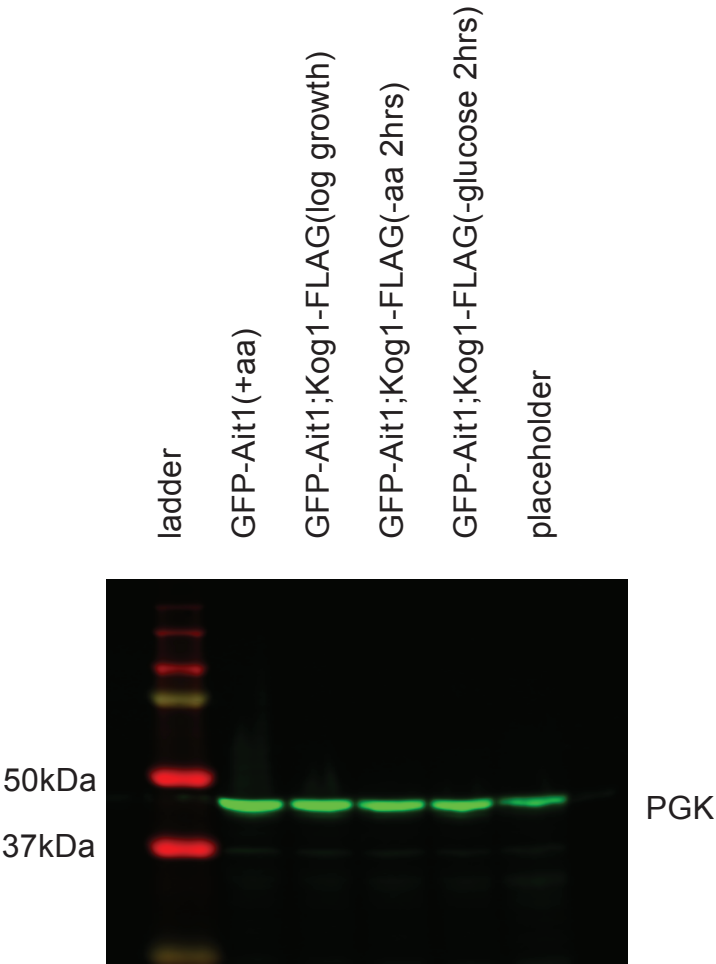

Supplement: Source data 1. — The original labeled gel, from each panel in Figure 3—figure supplement 1, Figure 6, Figure 6—figure supplement 1, Figure 7, Figure 8, Figure 8—figure supplement 3, Figure 9, and Figure 9—figure supplement 1 are included in source data in two separate folders. In each case the gels are numbered as they are shown in the associated figure—from top to bottom. In the case of Figure 6, the two gels on the left are labeled 1 and 2 and the two gels on the right are labeled 3 and 4. [file elife-68773-data1.zip › labelled gel figures/Figure3-figure supplement 1-source data 6 label.pdf]

Gtr1-Myc IPs +/- amino acids

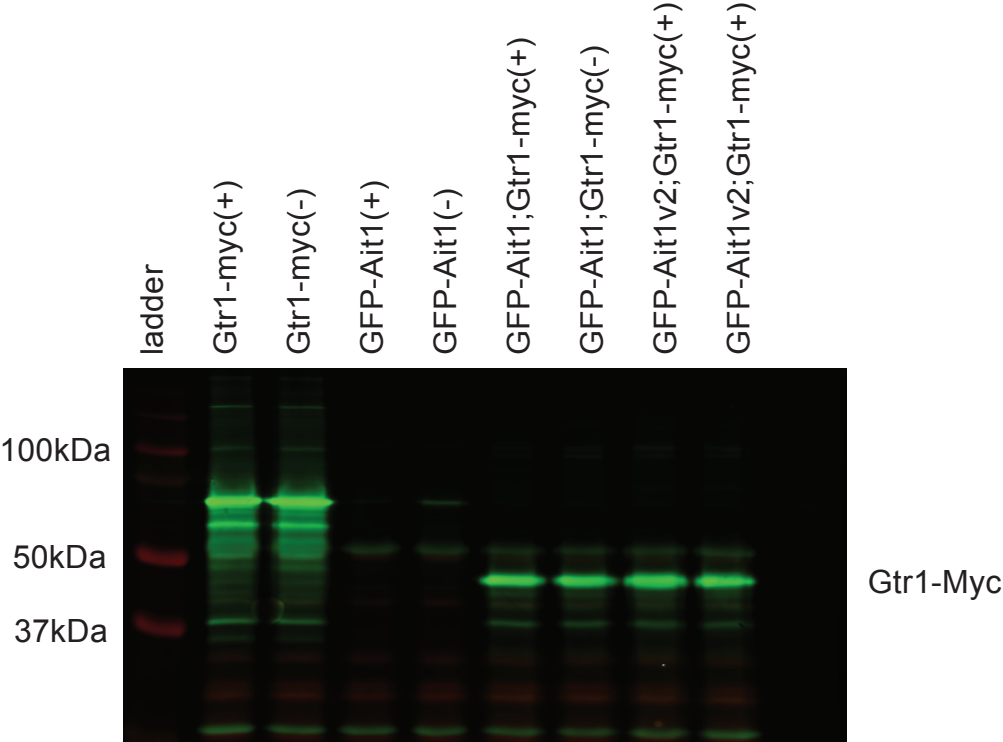

Supplement: Source data 1. — The original labeled gel, from each panel in Figure 3—figure supplement 1, Figure 6, Figure 6—figure supplement 1, Figure 7, Figure 8, Figure 8—figure supplement 3, Figure 9, and Figure 9—figure supplement 1 are included in source data in two separate folders. In each case the gels are numbered as they are shown in the associated figure—from top to bottom. In the case of Figure 6, the two gels on the left are labeled 1 and 2 and the two gels on the right are labeled 3 and 4. [file elife-68773-data1.zip › labelled gel figures/Figure9-source data 2 label.pdf]

leucine starvation

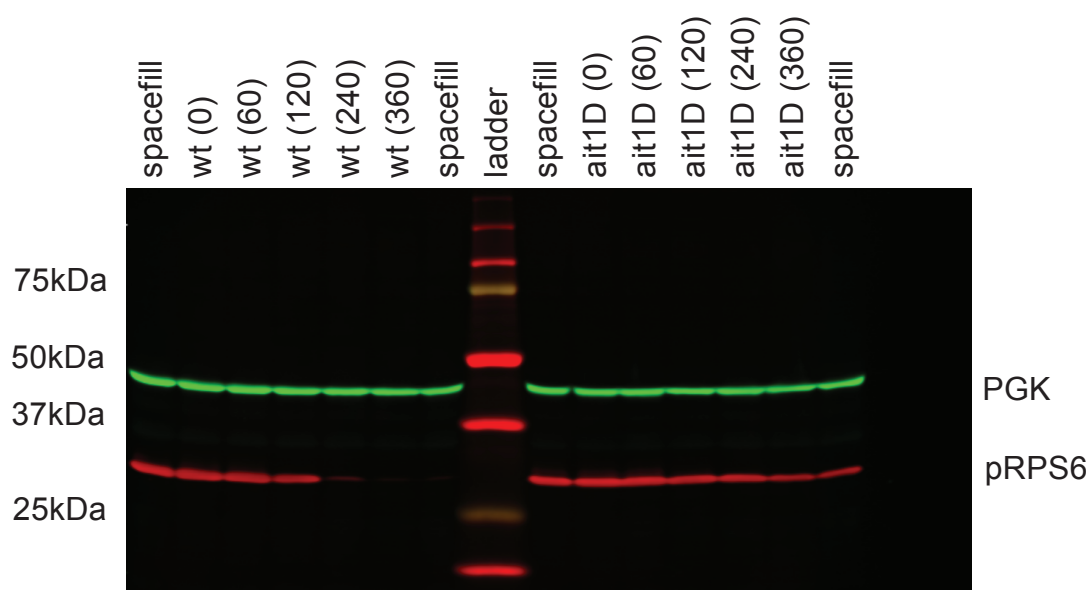

Supplement: Source data 1. — The original labeled gel, from each panel in Figure 3—figure supplement 1, Figure 6, Figure 6—figure supplement 1, Figure 7, Figure 8, Figure 8—figure supplement 3, Figure 9, and Figure 9—figure supplement 1 are included in source data in two separate folders. In each case the gels are numbered as they are shown in the associated figure—from top to bottom. In the case of Figure 6, the two gels on the left are labeled 1 and 2 and the two gels on the right are labeled 3 and 4. [file elife-68773-data1.zip › labelled gel figures/Figure6-source data 2 label.pdf]

# Gtr1/2 delete background

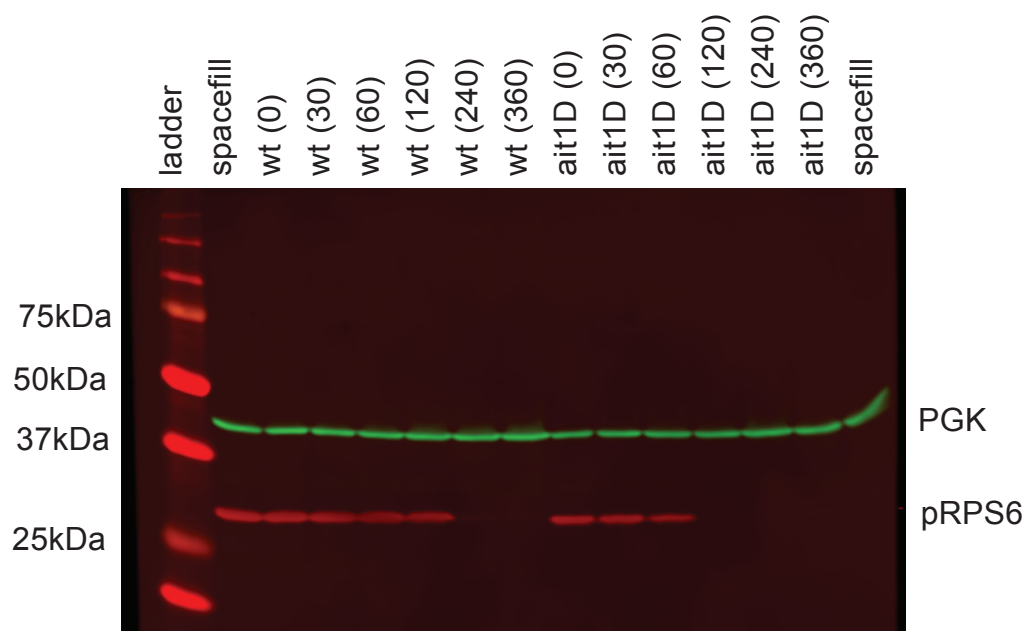

Supplement: Source data 1. — The original labeled gel, from each panel in Figure 3—figure supplement 1, Figure 6, Figure 6—figure supplement 1, Figure 7, Figure 8, Figure 8—figure supplement 3, Figure 9, and Figure 9—figure supplement 1 are included in source data in two separate folders. In each case the gels are numbered as they are shown in the associated figure—from top to bottom. In the case of Figure 6, the two gels on the left are labeled 1 and 2 and the two gels on the right are labeled 3 and 4. [file elife-68773-data1.zip › labelled gel figures/Figure7-source data 6 label.pdf]

PGK +/- nutrients

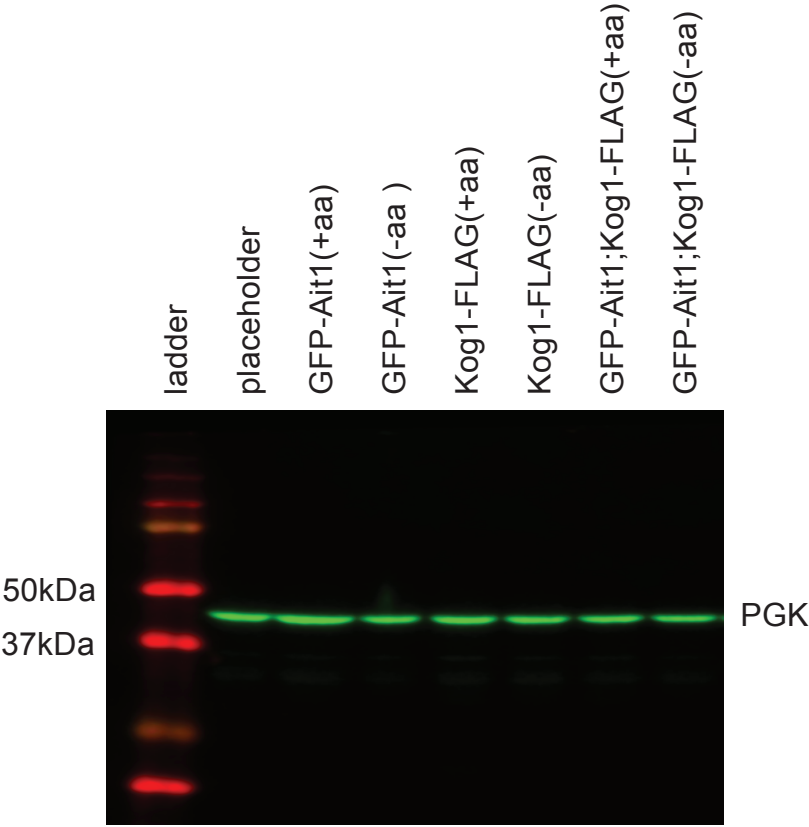

Supplement: Source data 1. — The original labeled gel, from each panel in Figure 3—figure supplement 1, Figure 6, Figure 6—figure supplement 1, Figure 7, Figure 8, Figure 8—figure supplement 3, Figure 9, and Figure 9—figure supplement 1 are included in source data in two separate folders. In each case the gels are numbered as they are shown in the associated figure—from top to bottom. In the case of Figure 6, the two gels on the left are labeled 1 and 2 and the two gels on the right are labeled 3 and 4. [file elife-68773-data1.zip › labelled gel figures/Figure3-figure supplement 1-source data 3 label.pdf]

## Ait1v2 mutant in amino acid starvation

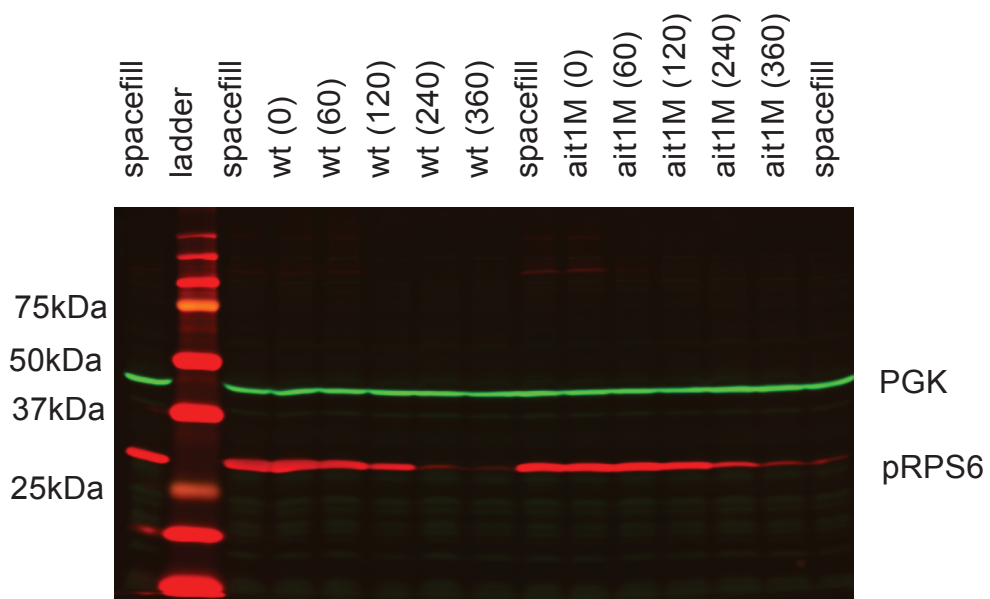

Supplement: Source data 1. — The original labeled gel, from each panel in Figure 3—figure supplement 1, Figure 6, Figure 6—figure supplement 1, Figure 7, Figure 8, Figure 8—figure supplement 3, Figure 9, and Figure 9—figure supplement 1 are included in source data in two separate folders. In each case the gels are numbered as they are shown in the associated figure—from top to bottom. In the case of Figure 6, the two gels on the left are labeled 1 and 2 and the two gels on the right are labeled 3 and 4. [file elife-68773-data1.zip › labelled gel figures/Figure8-figure supplement 3-source data 2 label.pdf]

# Gtr1/2off background

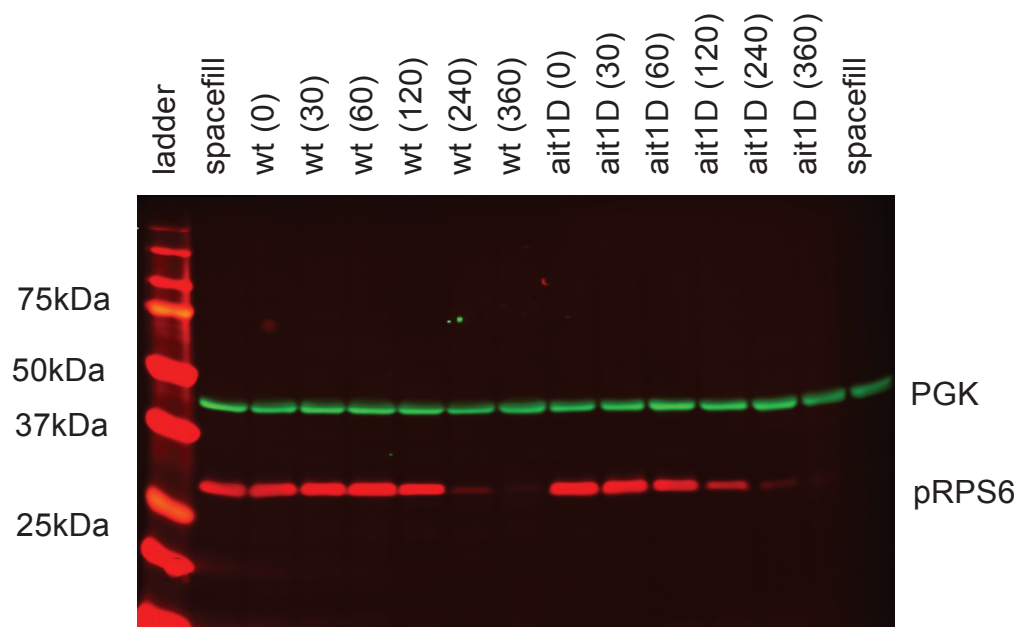

Supplement: Source data 1. — The original labeled gel, from each panel in Figure 3—figure supplement 1, Figure 6, Figure 6—figure supplement 1, Figure 7, Figure 8, Figure 8—figure supplement 3, Figure 9, and Figure 9—figure supplement 1 are included in source data in two separate folders. In each case the gels are numbered as they are shown in the associated figure—from top to bottom. In the case of Figure 6, the two gels on the left are labeled 1 and 2 and the two gels on the right are labeled 3 and 4. [file elife-68773-data1.zip › labelled gel figures/Figure7-source data 3 label.pdf]

# Ait1v1 mutant

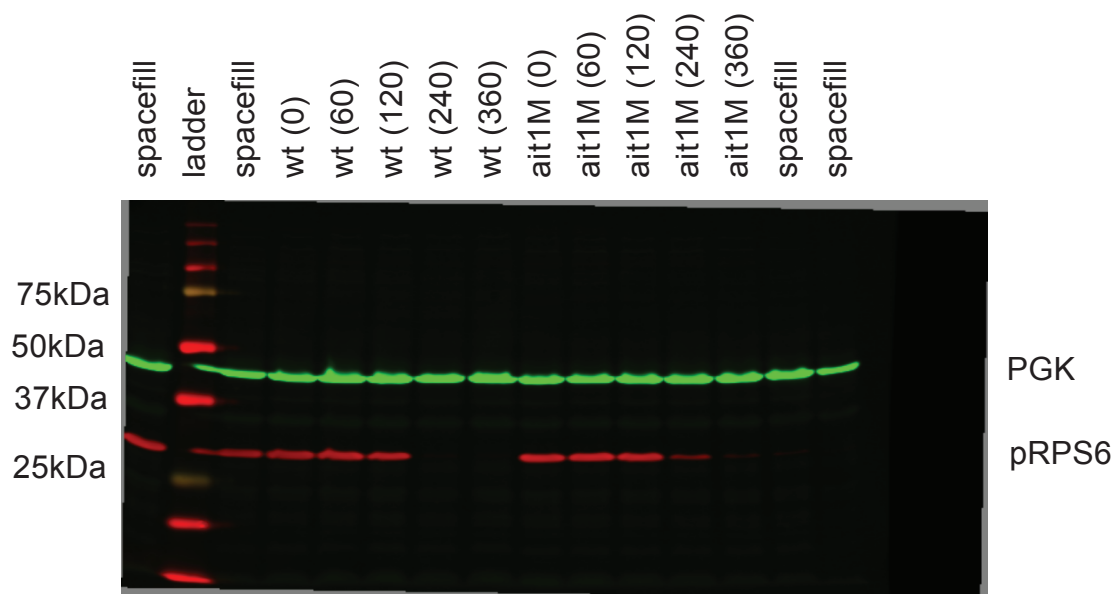

Supplement: Source data 1. — The original labeled gel, from each panel in Figure 3—figure supplement 1, Figure 6, Figure 6—figure supplement 1, Figure 7, Figure 8, Figure 8—figure supplement 3, Figure 9, and Figure 9—figure supplement 1 are included in source data in two separate folders. In each case the gels are numbered as they are shown in the associated figure—from top to bottom. In the case of Figure 6, the two gels on the left are labeled 1 and 2 and the two gels on the right are labeled 3 and 4. [file elife-68773-data1.zip › labelled gel figures/Figure8-source data 3 label.pdf]

## amino acid starvation

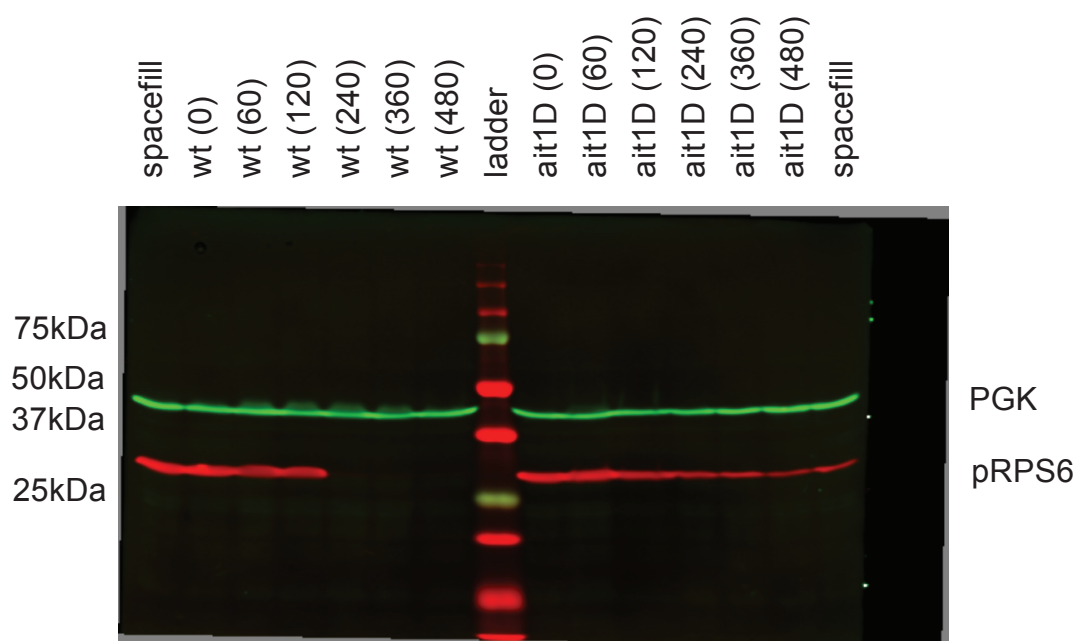

Supplement: Source data 1. — The original labeled gel, from each panel in Figure 3—figure supplement 1, Figure 6, Figure 6—figure supplement 1, Figure 7, Figure 8, Figure 8—figure supplement 3, Figure 9, and Figure 9—figure supplement 1 are included in source data in two separate folders. In each case the gels are numbered as they are shown in the associated figure—from top to bottom. In the case of Figure 6, the two gels on the left are labeled 1 and 2 and the two gels on the right are labeled 3 and 4. [file elife-68773-data1.zip › labelled gel figures/Figure6-source data 1 label.pdf]

GFP-Ait1 IPs +/- nutrients

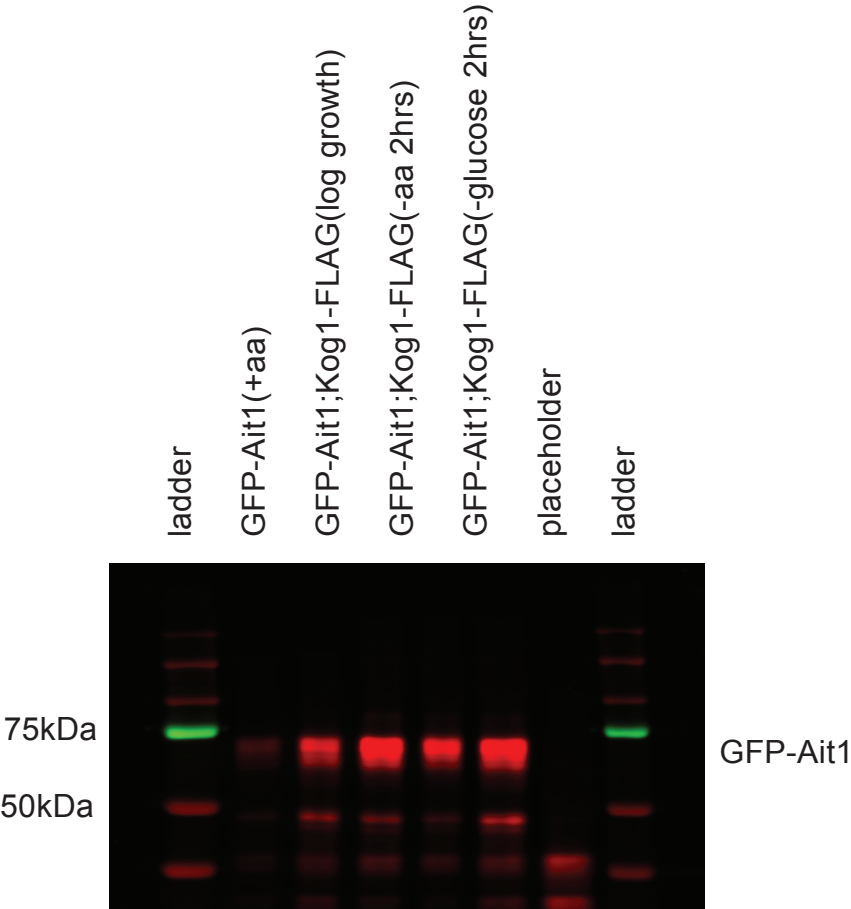

Supplement: Source data 1. — The original labeled gel, from each panel in Figure 3—figure supplement 1, Figure 6, Figure 6—figure supplement 1, Figure 7, Figure 8, Figure 8—figure supplement 3, Figure 9, and Figure 9—figure supplement 1 are included in source data in two separate folders. In each case the gels are numbered as they are shown in the associated figure—from top to bottom. In the case of Figure 6, the two gels on the left are labeled 1 and 2 and the two gels on the right are labeled 3 and 4. [file elife-68773-data1.zip › labelled gel figures/Figure3-figure supplement 1-source data 5 label.pdf]

GFP-Ait1 IPs +/- amino acids

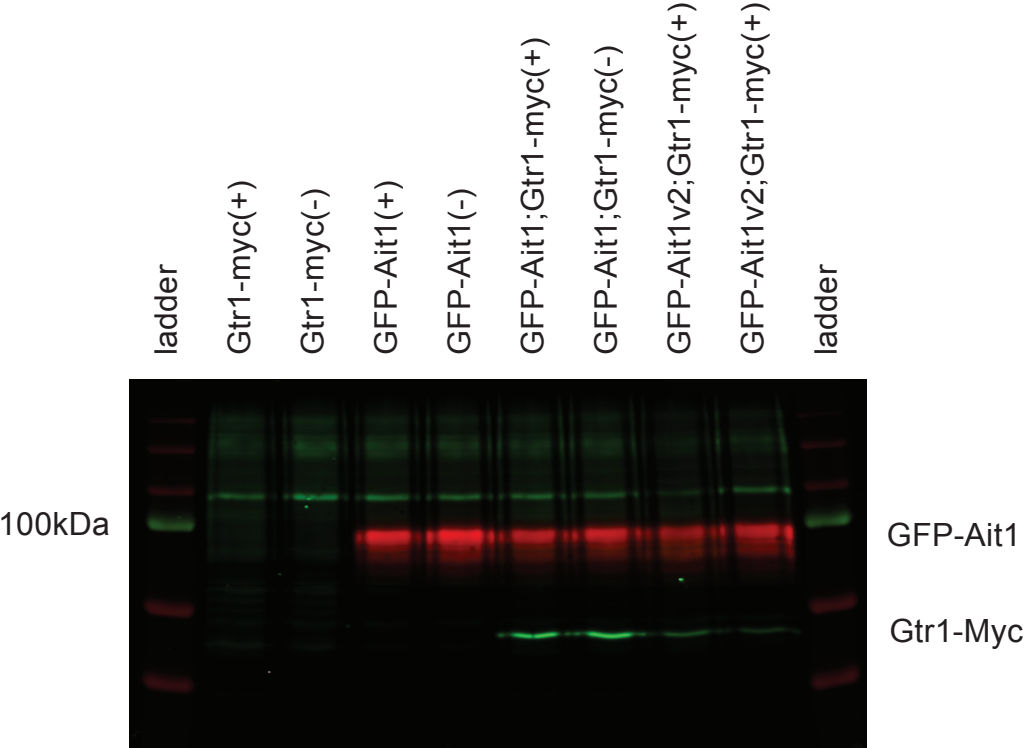

Supplement: Source data 1. — The original labeled gel, from each panel in Figure 3—figure supplement 1, Figure 6, Figure 6—figure supplement 1, Figure 7, Figure 8, Figure 8—figure supplement 3, Figure 9, and Figure 9—figure supplement 1 are included in source data in two separate folders. In each case the gels are numbered as they are shown in the associated figure—from top to bottom. In the case of Figure 6, the two gels on the left are labeled 1 and 2 and the two gels on the right are labeled 3 and 4. [file elife-68773-data1.zip › labelled gel figures/Figure9-source data 1 label.pdf]

## Ait1v3 mutant

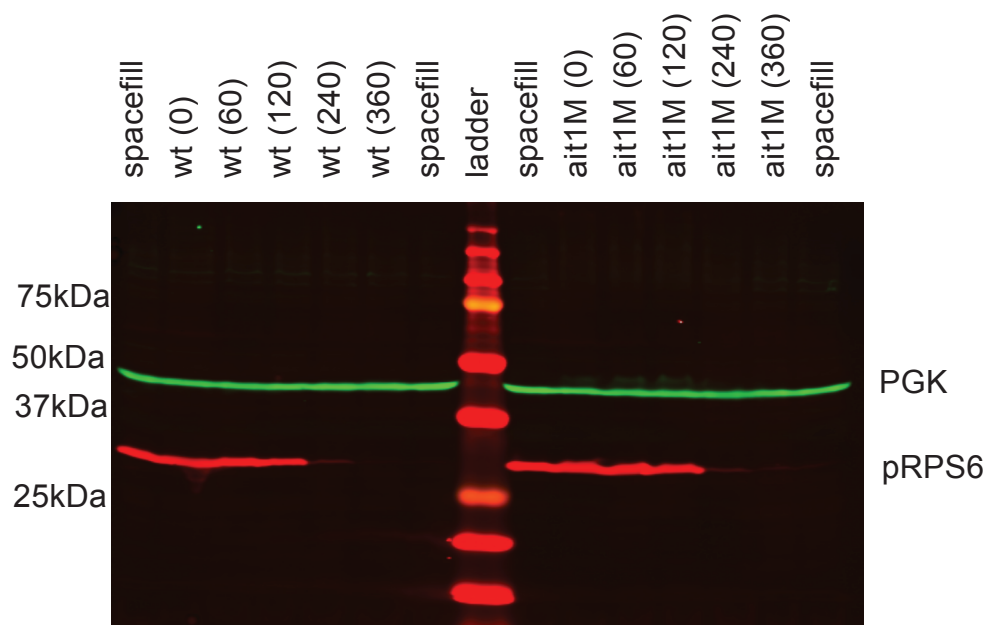

Supplement: Source data 1. — The original labeled gel, from each panel in Figure 3—figure supplement 1, Figure 6, Figure 6—figure supplement 1, Figure 7, Figure 8, Figure 8—figure supplement 3, Figure 9, and Figure 9—figure supplement 1 are included in source data in two separate folders. In each case the gels are numbered as they are shown in the associated figure—from top to bottom. In the case of Figure 6, the two gels on the left are labeled 1 and 2 and the two gels on the right are labeled 3 and 4. [file elife-68773-data1.zip › labelled gel figures/Figure8-source data 5 label.pdf]

## Gtr2 delete background

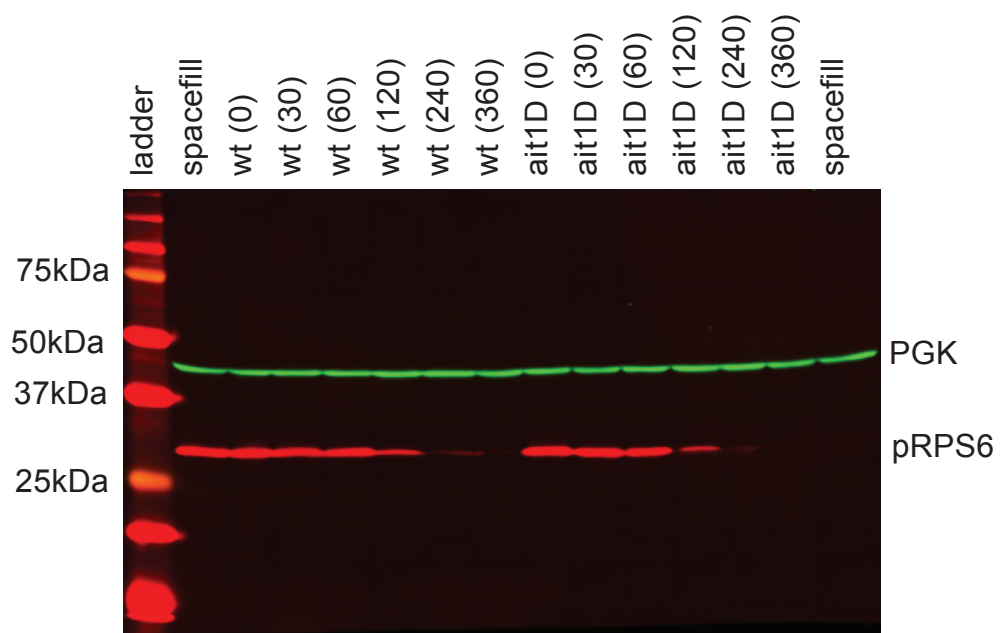

Supplement: Source data 1. — The original labeled gel, from each panel in Figure 3—figure supplement 1, Figure 6, Figure 6—figure supplement 1, Figure 7, Figure 8, Figure 8—figure supplement 3, Figure 9, and Figure 9—figure supplement 1 are included in source data in two separate folders. In each case the gels are numbered as they are shown in the associated figure—from top to bottom. In the case of Figure 6, the two gels on the left are labeled 1 and 2 and the two gels on the right are labeled 3 and 4. [file elife-68773-data1.zip › labelled gel figures/Figure7-source data 5 label.pdf]

## Ait1v2 mutant in nitrogen starvation

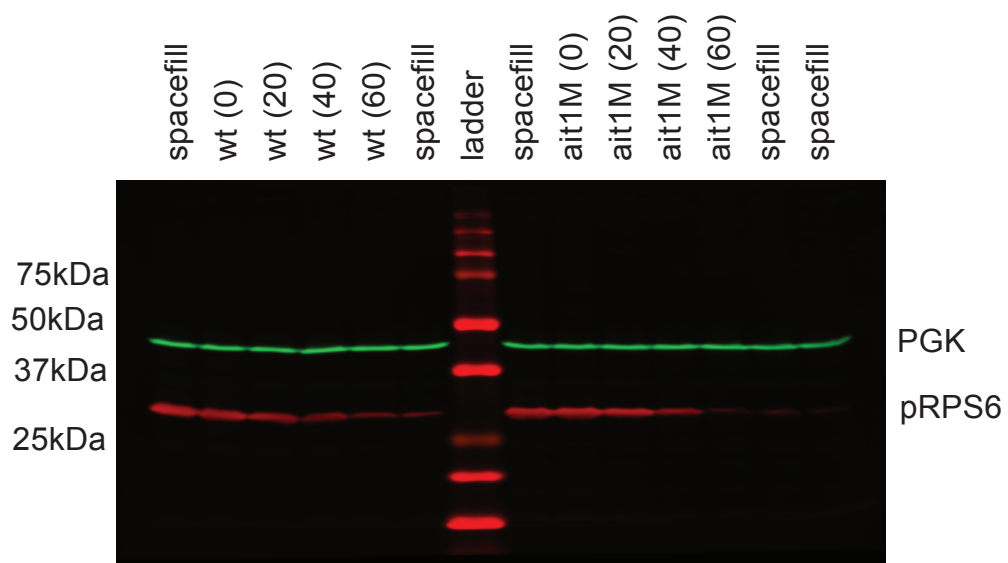

Supplement: Source data 1. — The original labeled gel, from each panel in Figure 3—figure supplement 1, Figure 6, Figure 6—figure supplement 1, Figure 7, Figure 8, Figure 8—figure supplement 3, Figure 9, and Figure 9—figure supplement 1 are included in source data in two separate folders. In each case the gels are numbered as they are shown in the associated figure—from top to bottom. In the case of Figure 6, the two gels on the left are labeled 1 and 2 and the two gels on the right are labeled 3 and 4. [file elife-68773-data1.zip › labelled gel figures/Figure8-figure supplement 3-source data 3 label.pdf]

GFP-Ait1 IPs +/- nutrients

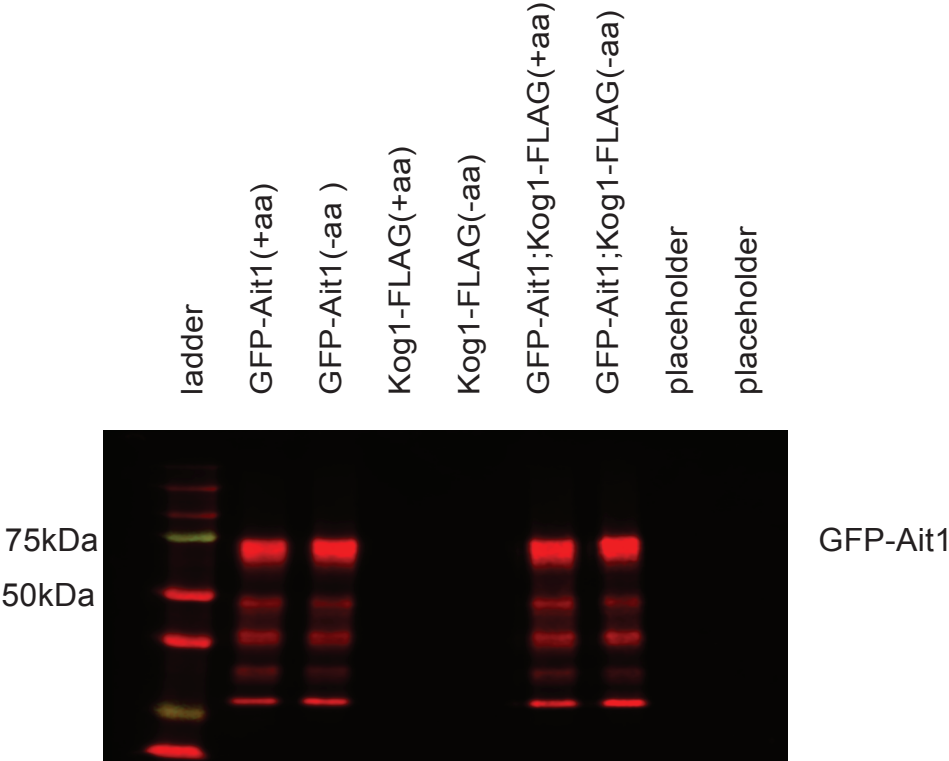

Supplement: Source data 1. — The original labeled gel, from each panel in Figure 3—figure supplement 1, Figure 6, Figure 6—figure supplement 1, Figure 7, Figure 8, Figure 8—figure supplement 3, Figure 9, and Figure 9—figure supplement 1 are included in source data in two separate folders. In each case the gels are numbered as they are shown in the associated figure—from top to bottom. In the case of Figure 6, the two gels on the left are labeled 1 and 2 and the two gels on the right are labeled 3 and 4. [file elife-68773-data1.zip › labelled gel figures/Figure3-figure supplement 1-source data 2 label.pdf]

## Gtr2off background

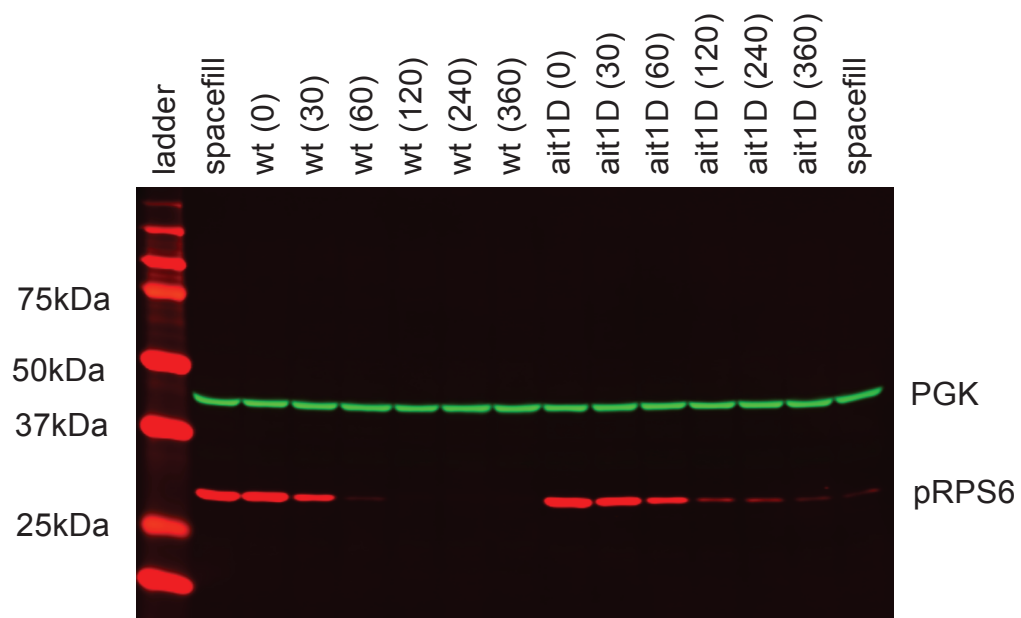

Supplement: Source data 1. — The original labeled gel, from each panel in Figure 3—figure supplement 1, Figure 6, Figure 6—figure supplement 1, Figure 7, Figure 8, Figure 8—figure supplement 3, Figure 9, and Figure 9—figure supplement 1 are included in source data in two separate folders. In each case the gels are numbered as they are shown in the associated figure—from top to bottom. In the case of Figure 6, the two gels on the left are labeled 1 and 2 and the two gels on the right are labeled 3 and 4. [file elife-68773-data1.zip › labelled gel figures/Figure7-source data 2 label.pdf]

## Ait1DC3 mutant

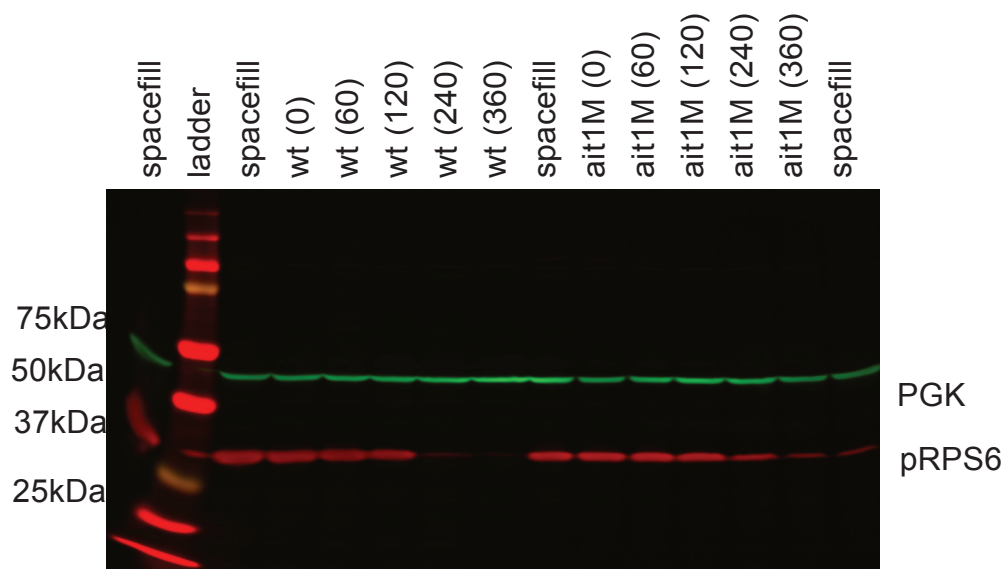

Supplement: Source data 1. — The original labeled gel, from each panel in Figure 3—figure supplement 1, Figure 6, Figure 6—figure supplement 1, Figure 7, Figure 8, Figure 8—figure supplement 3, Figure 9, and Figure 9—figure supplement 1 are included in source data in two separate folders. In each case the gels are numbered as they are shown in the associated figure—from top to bottom. In the case of Figure 6, the two gels on the left are labeled 1 and 2 and the two gels on the right are labeled 3 and 4. [file elife-68773-data1.zip › labelled gel figures/Figure8-source data 2 label.pdf]

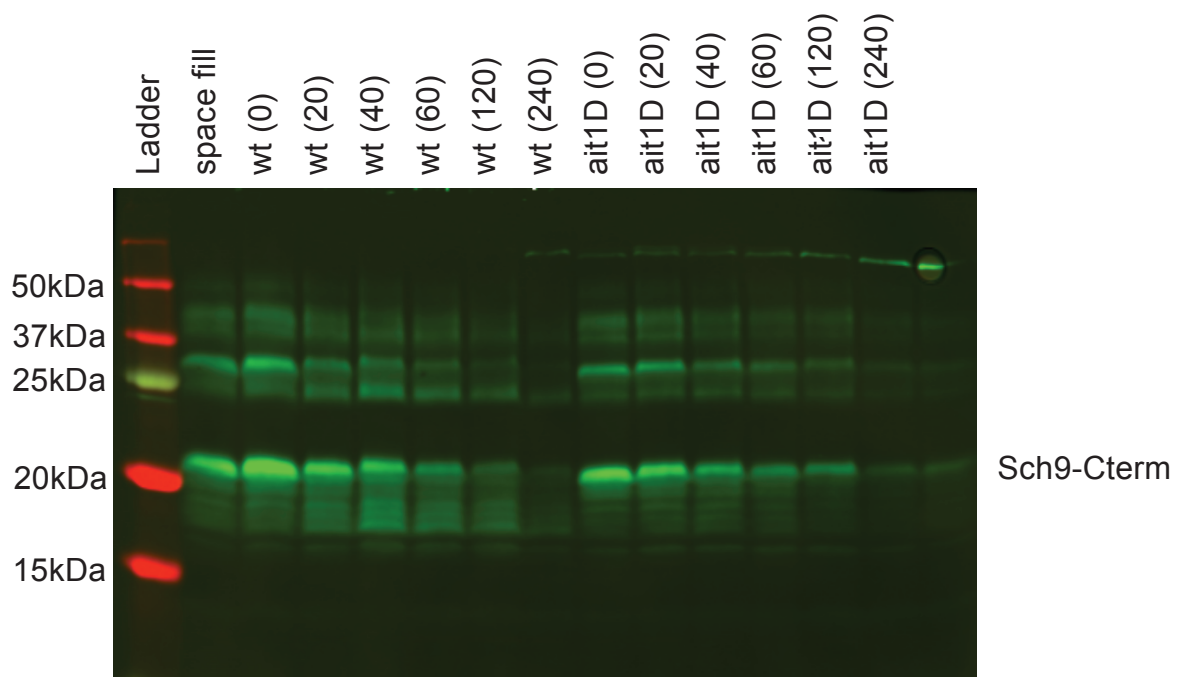

Supplement: Source data 1. — The original labeled gel, from each panel in Figure 3—figure supplement 1, Figure 6, Figure 6—figure supplement 1, Figure 7, Figure 8, Figure 8—figure supplement 3, Figure 9, and Figure 9—figure supplement 1 are included in source data in two separate folders. In each case the gels are numbered as they are shown in the associated figure—from top to bottom. In the case of Figure 6, the two gels on the left are labeled 1 and 2 and the two gels on the right are labeled 3 and 4. [file elife-68773-data1.zip › labelled gel figures/Figure6-figure supplement 1-source data 2 label.pdf]

Kog1-FLAG IPs +/- nutrients

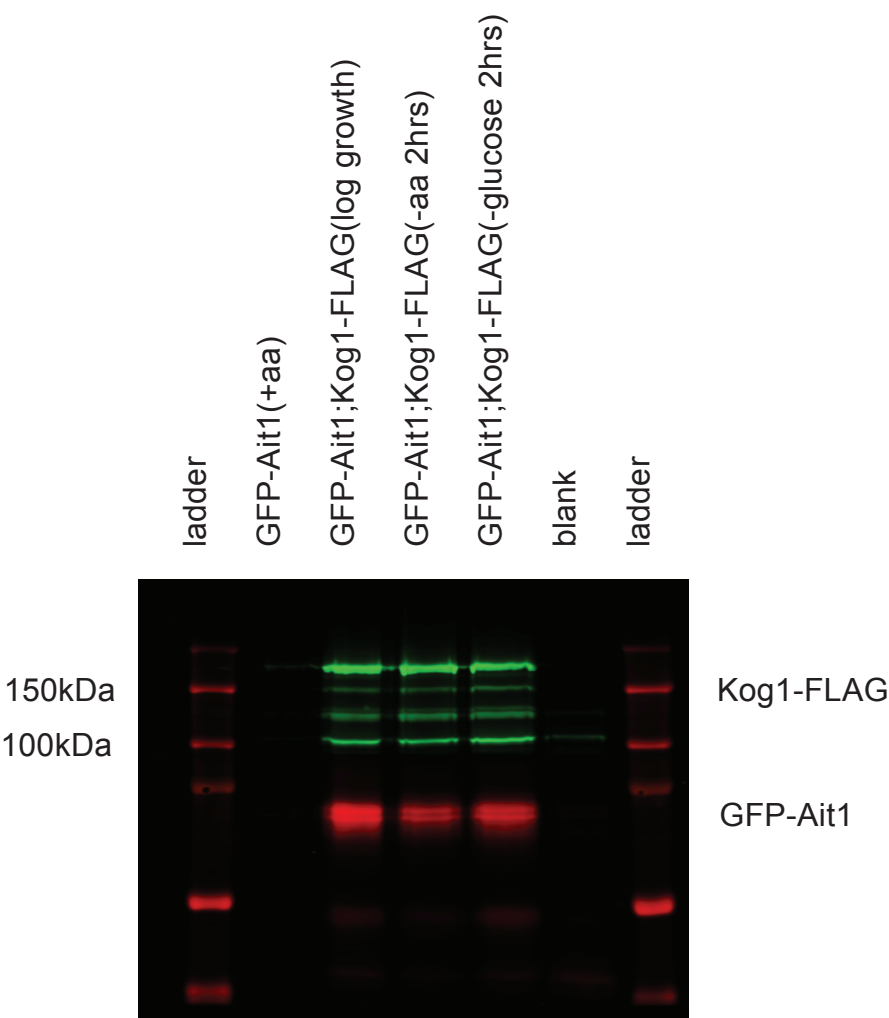

Supplement: Source data 1. — The original labeled gel, from each panel in Figure 3—figure supplement 1, Figure 6, Figure 6—figure supplement 1, Figure 7, Figure 8, Figure 8—figure supplement 3, Figure 9, and Figure 9—figure supplement 1 are included in source data in two separate folders. In each case the gels are numbered as they are shown in the associated figure—from top to bottom. In the case of Figure 6, the two gels on the left are labeled 1 and 2 and the two gels on the right are labeled 3 and 4. [file elife-68773-data1.zip › labelled gel figures/Figure3-figure supplement 1-source data 4 label.pdf]

## Ait1v2 mutant

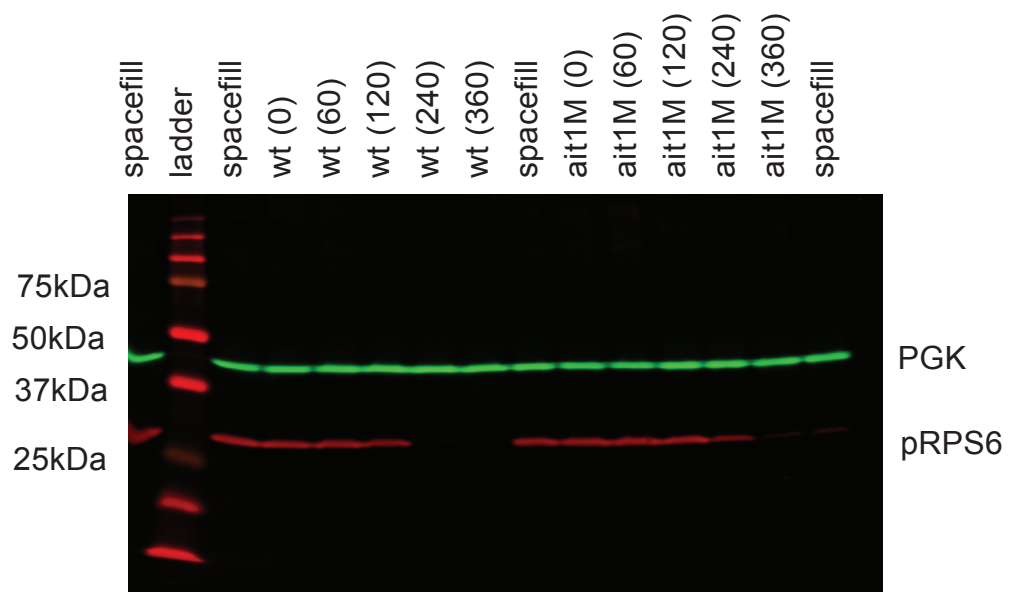

Supplement: Source data 1. — The original labeled gel, from each panel in Figure 3—figure supplement 1, Figure 6, Figure 6—figure supplement 1, Figure 7, Figure 8, Figure 8—figure supplement 3, Figure 9, and Figure 9—figure supplement 1 are included in source data in two separate folders. In each case the gels are numbered as they are shown in the associated figure—from top to bottom. In the case of Figure 6, the two gels on the left are labeled 1 and 2 and the two gels on the right are labeled 3 and 4. [file elife-68773-data1.zip › labelled gel figures/Figure8-source data 4 label.pdf]

GFP-Ait1 IPs +/- nutrients

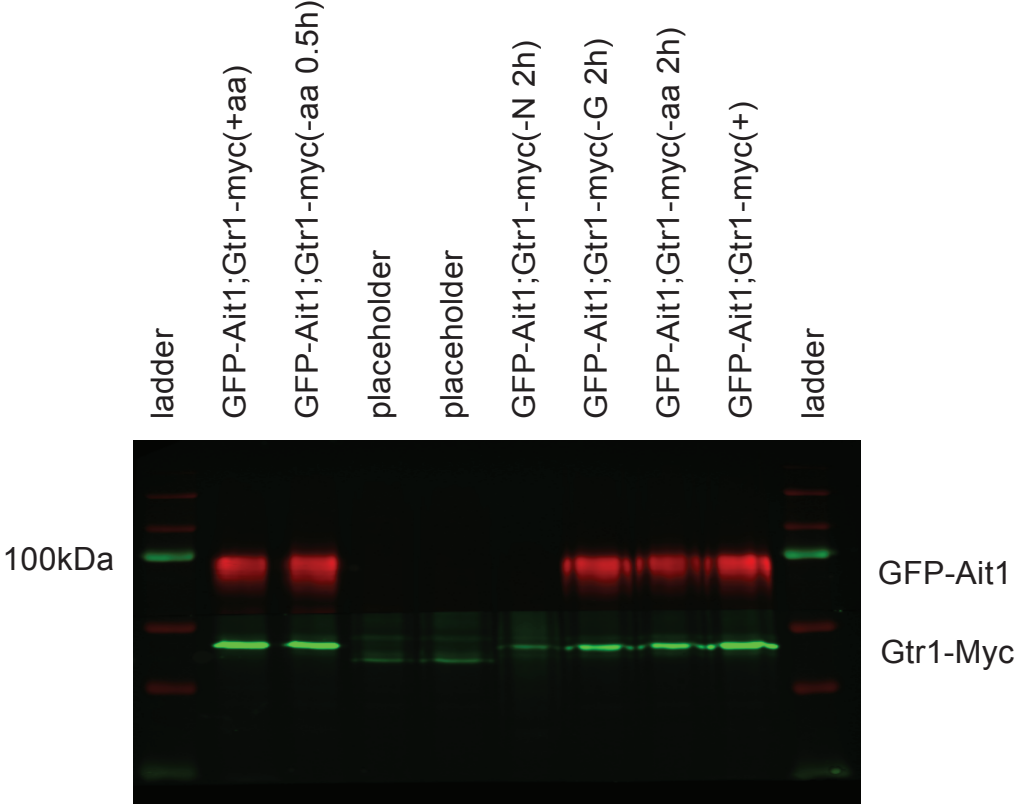

Supplement: Source data 1. — The original labeled gel, from each panel in Figure 3—figure supplement 1, Figure 6, Figure 6—figure supplement 1, Figure 7, Figure 8, Figure 8—figure supplement 3, Figure 9, and Figure 9—figure supplement 1 are included in source data in two separate folders. In each case the gels are numbered as they are shown in the associated figure—from top to bottom. In the case of Figure 6, the two gels on the left are labeled 1 and 2 and the two gels on the right are labeled 3 and 4. [file elife-68773-data1.zip › labelled gel figures/Figure9-figure supplement 1-source data 2 label.pdf]

## Gtr1 delete background

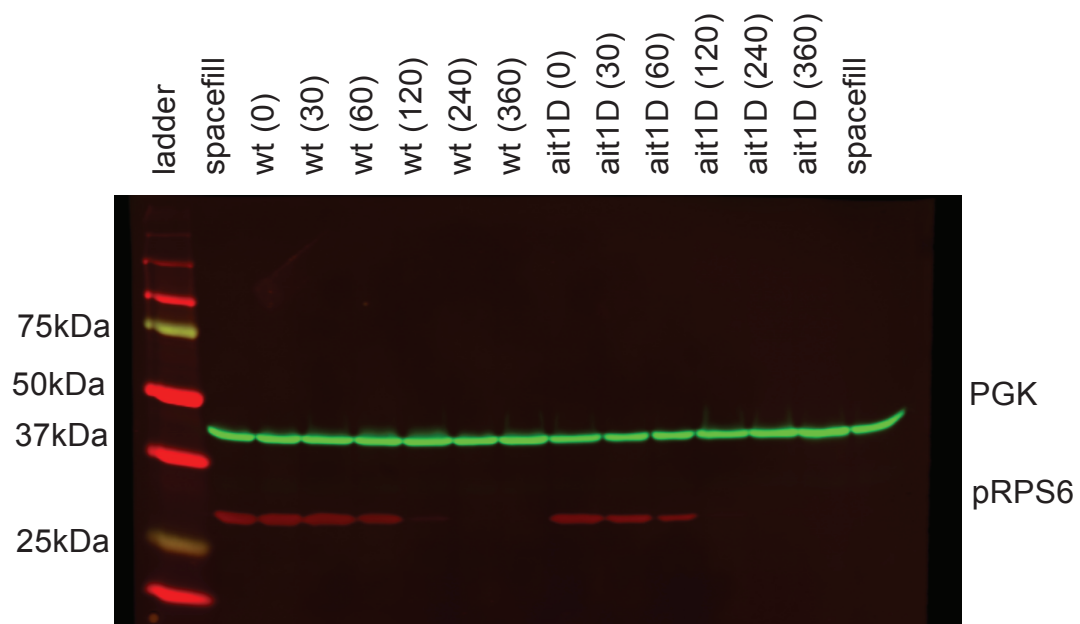

Supplement: Source data 1. — The original labeled gel, from each panel in Figure 3—figure supplement 1, Figure 6, Figure 6—figure supplement 1, Figure 7, Figure 8, Figure 8—figure supplement 3, Figure 9, and Figure 9—figure supplement 1 are included in source data in two separate folders. In each case the gels are numbered as they are shown in the associated figure—from top to bottom. In the case of Figure 6, the two gels on the left are labeled 1 and 2 and the two gels on the right are labeled 3 and 4. [file elife-68773-data1.zip › labelled gel figures/Figure7-source data 4 label.pdf]

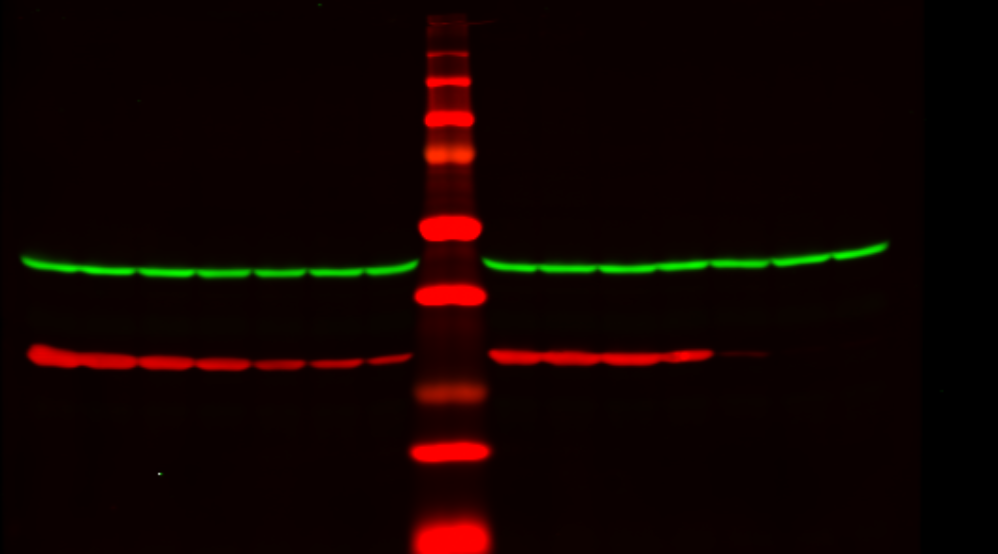

Supplement: Source data 2. [file elife-68773-data2.zip › raw gel images/Figure 7-source data 1.tif]

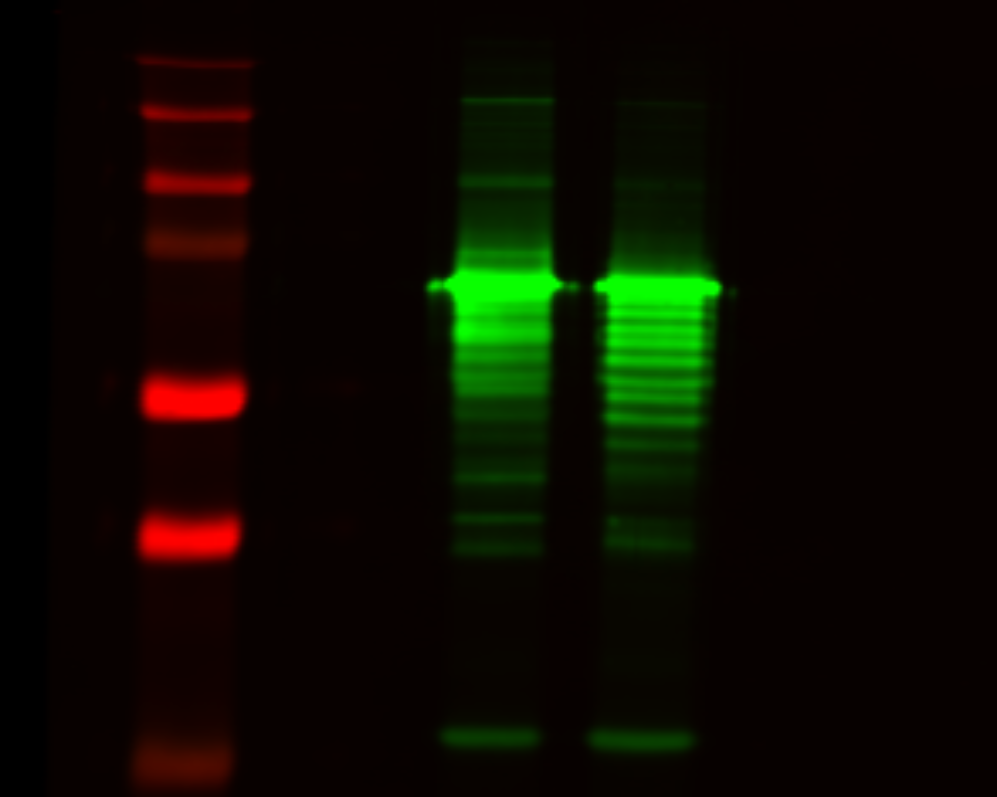

Supplement: Source data 2. [file elife-68773-data2.zip › raw gel images/Figure 9-figure supplement 1-source data 1.tif]

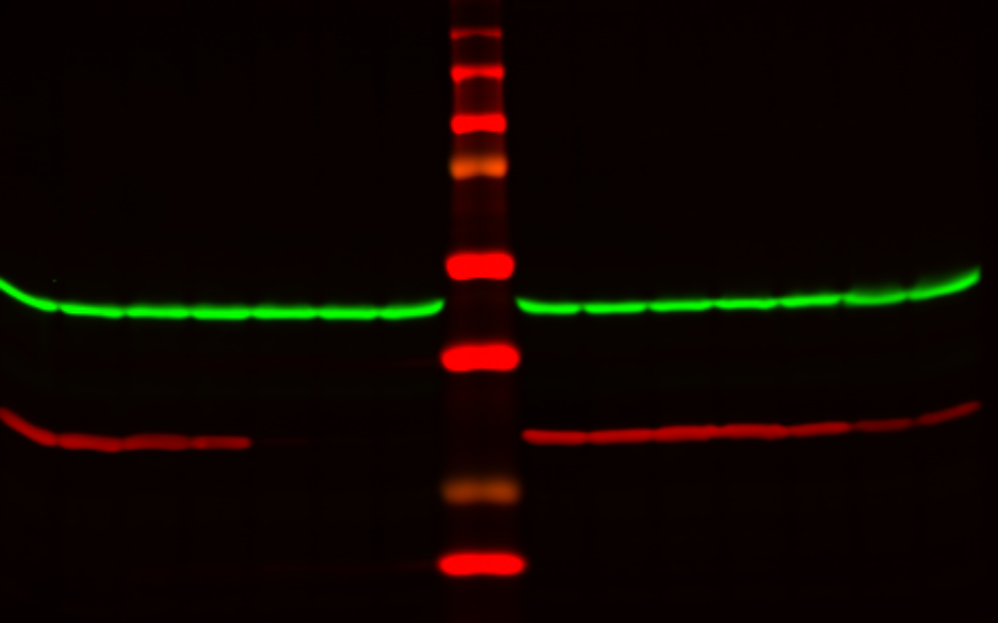

Supplement: Source data 2. [file elife-68773-data2.zip › raw gel images/Figure 6-source data 4.tif]

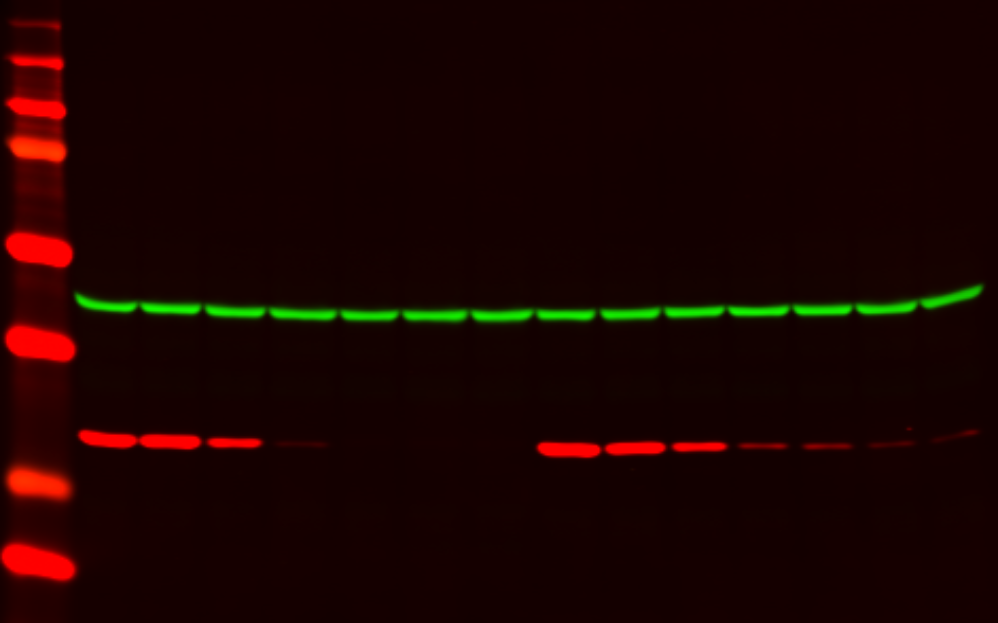

Supplement: Source data 2. [file elife-68773-data2.zip › raw gel images/Figure 7-source data 2.tif]

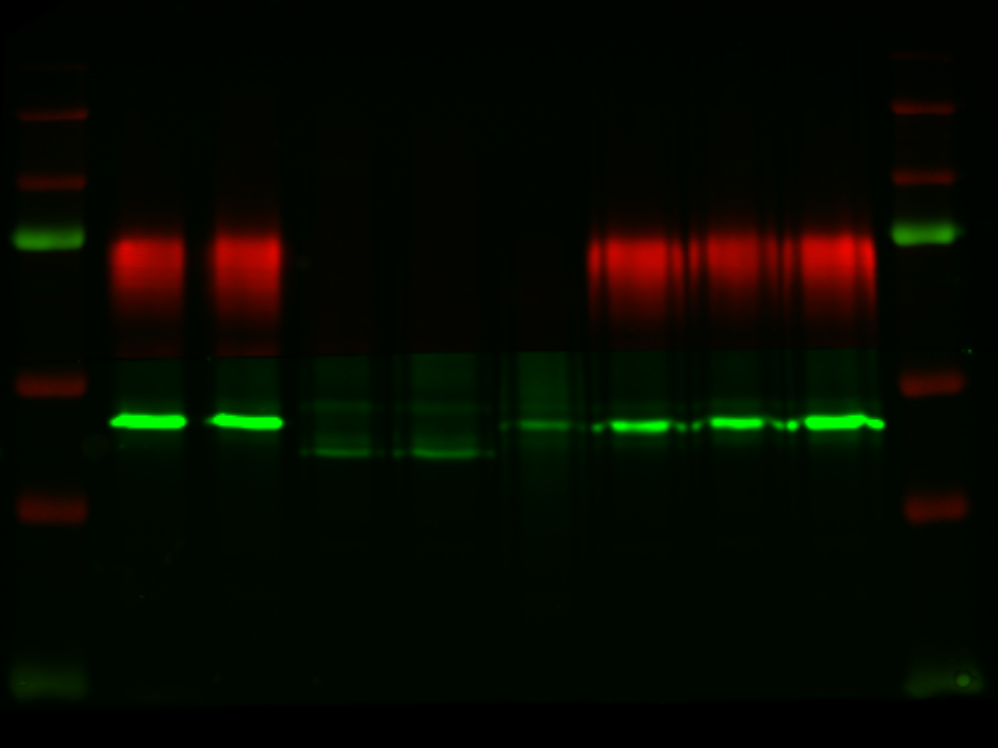

Supplement: Source data 2. [file elife-68773-data2.zip › raw gel images/Figure 9-figure supplement 1-source data 2.tif]

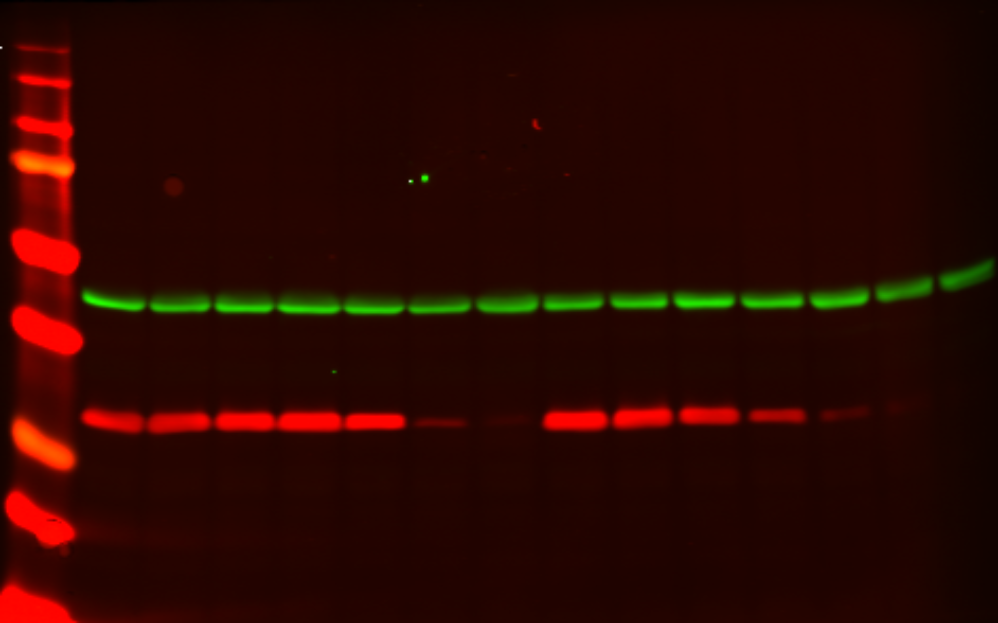

Supplement: Source data 2. [file elife-68773-data2.zip › raw gel images/Figure 7-source data 3.tif]

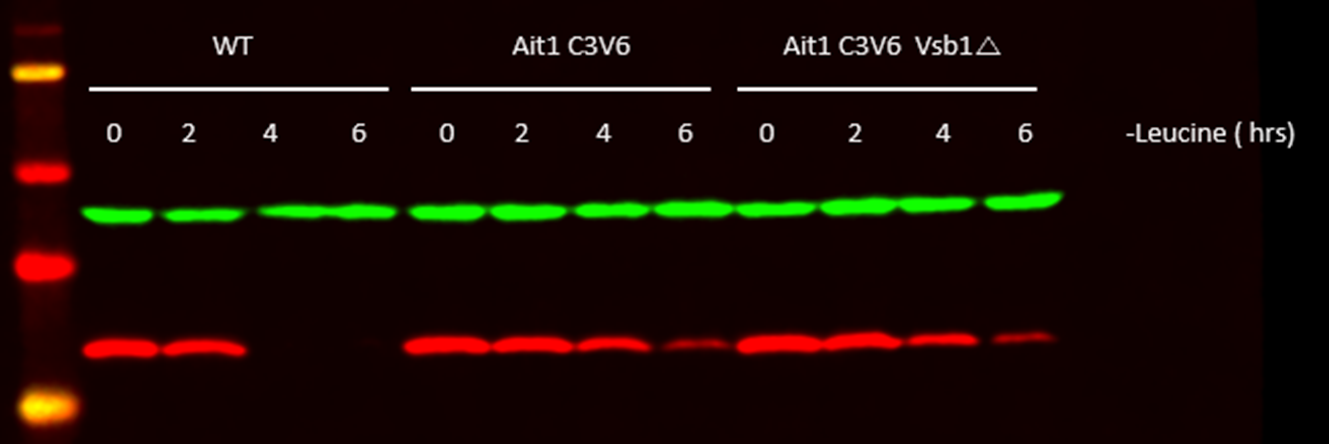

Supplement: Source data 2. [file elife-68773-data2.zip › raw gel images/Figure 8-figure supplement 3-source data 1.png]

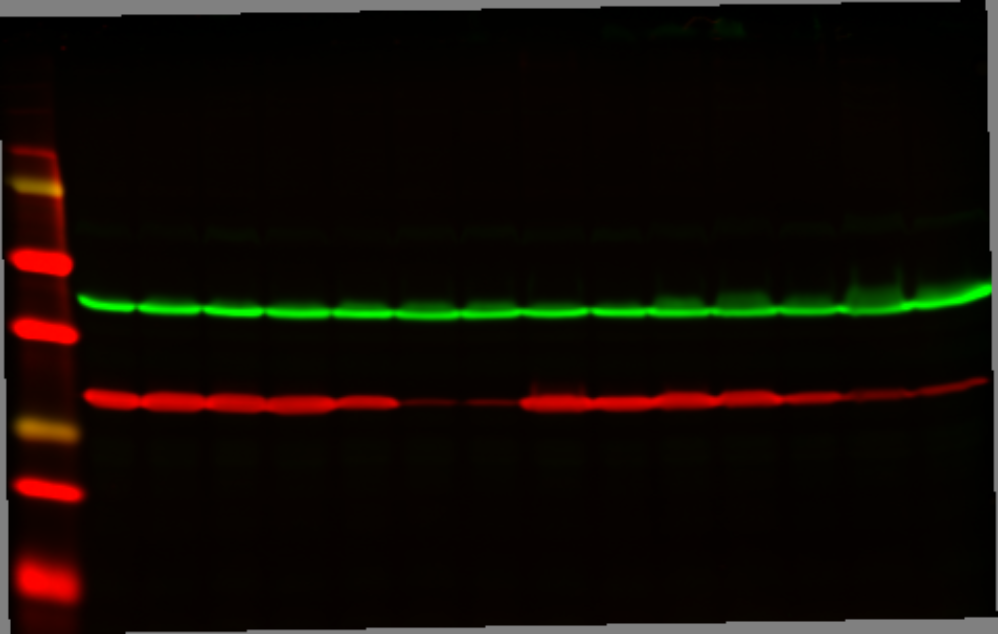

Supplement: Source data 2. [file elife-68773-data2.zip › raw gel images/Figure 7-source data 7.tif]

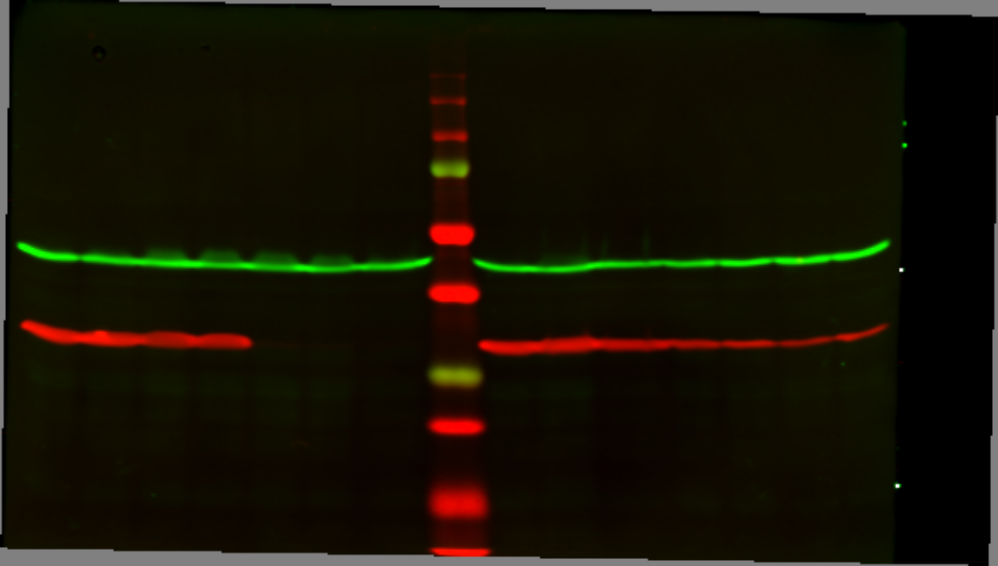

Supplement: Source data 2. [file elife-68773-data2.zip › raw gel images/Figure 6-source data 1.tif]

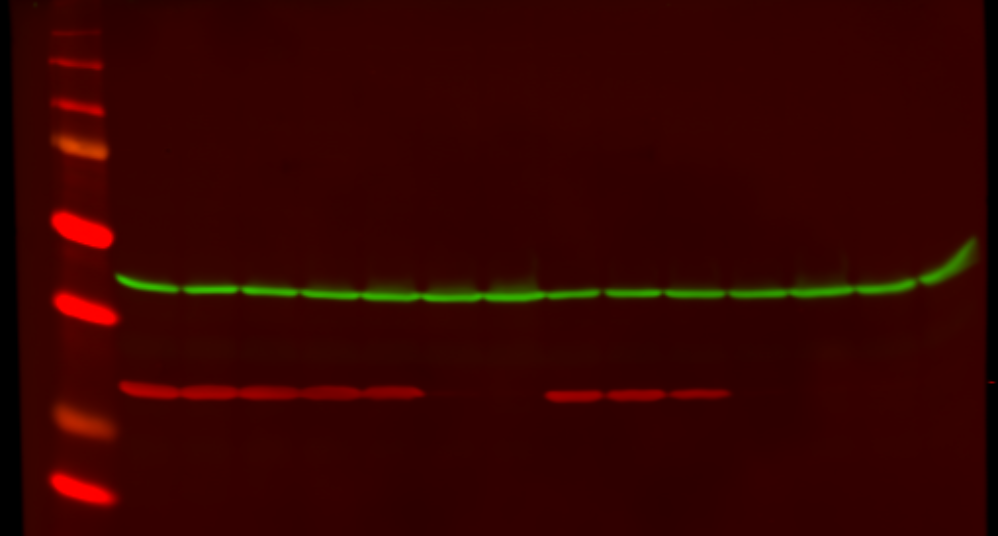

Supplement: Source data 2. [file elife-68773-data2.zip › raw gel images/Figure 7-source data 6.tif]

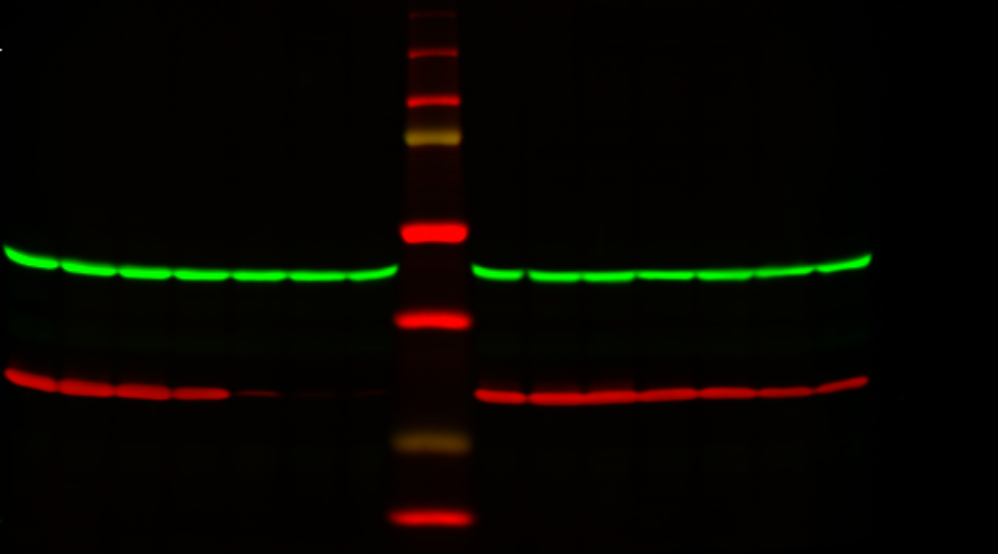

Supplement: Source data 2. [file elife-68773-data2.zip › raw gel images/Figure 6-source data 2.tif]

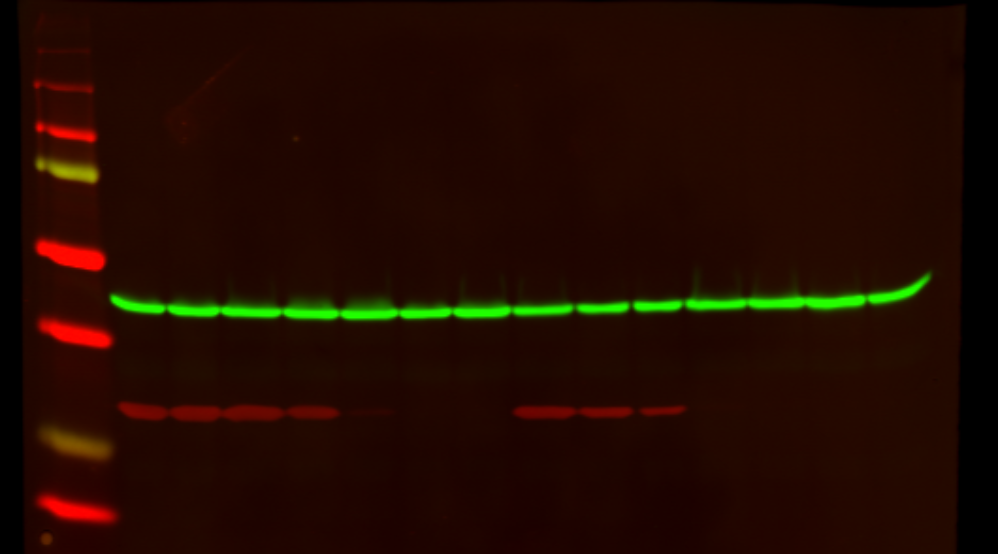

Supplement: Source data 2. [file elife-68773-data2.zip › raw gel images/Figure 7-source data 4.tif]

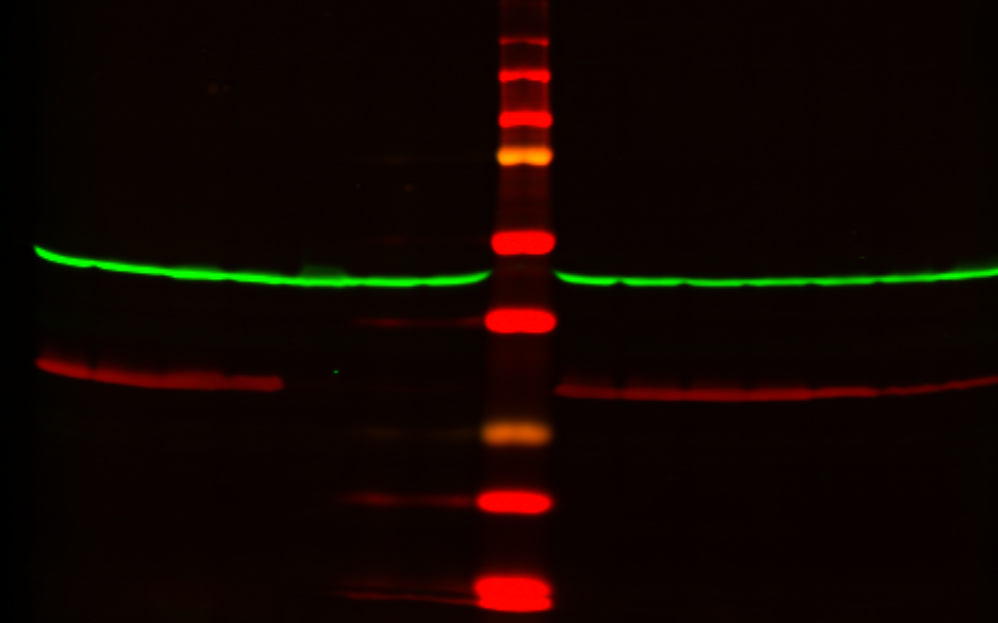

Supplement: Source data 2. [file elife-68773-data2.zip › raw gel images/Figure 6-source data 3.tif]

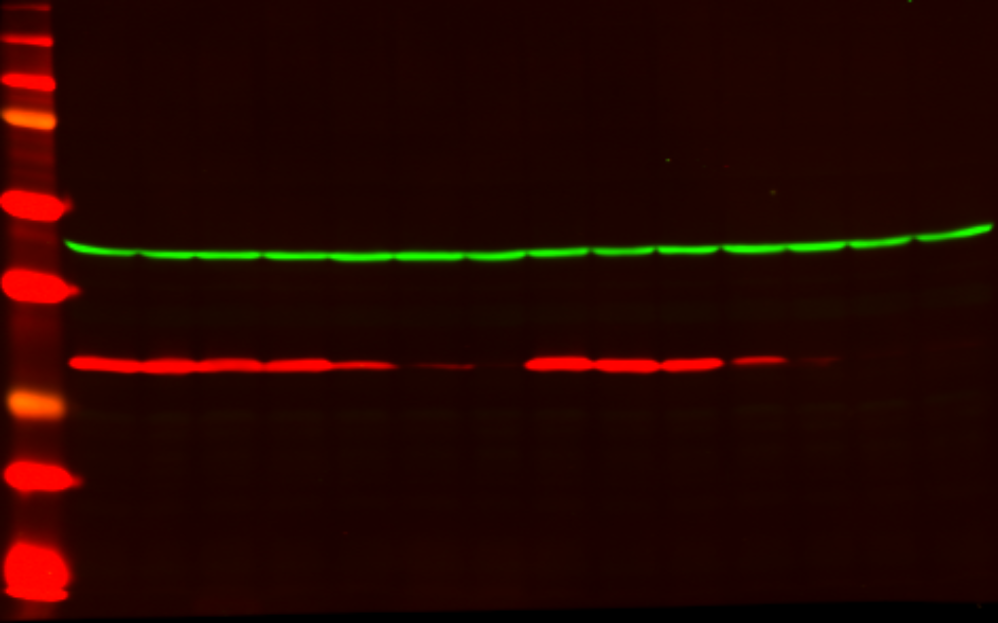

Supplement: Source data 2. [file elife-68773-data2.zip › raw gel images/Figure 7-source data 5.tif]

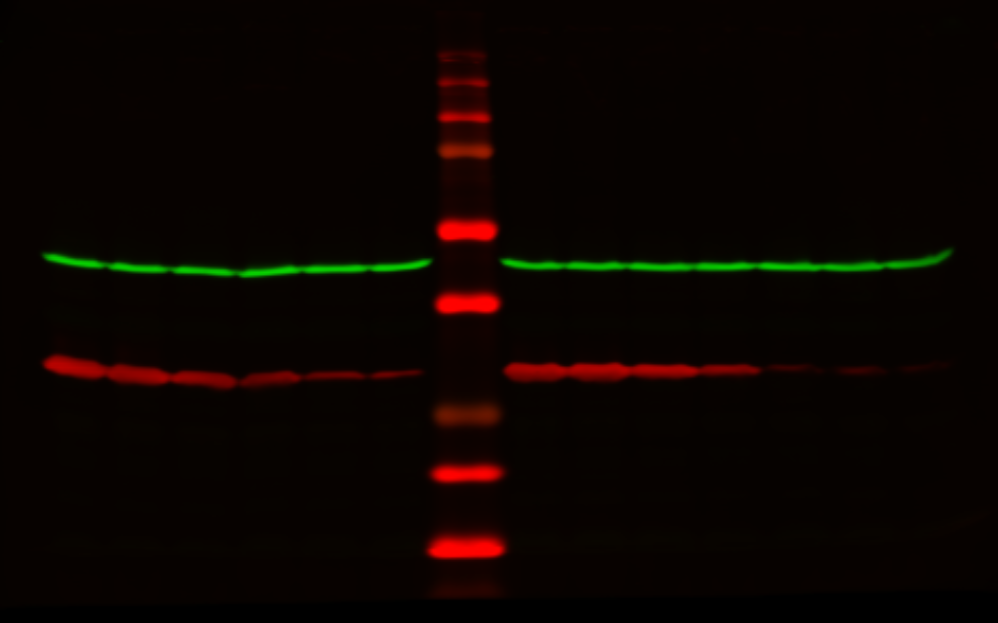

Supplement: Source data 2. [file elife-68773-data2.zip › raw gel images/Figure 8-figure supplement 3-source data 3.tif]

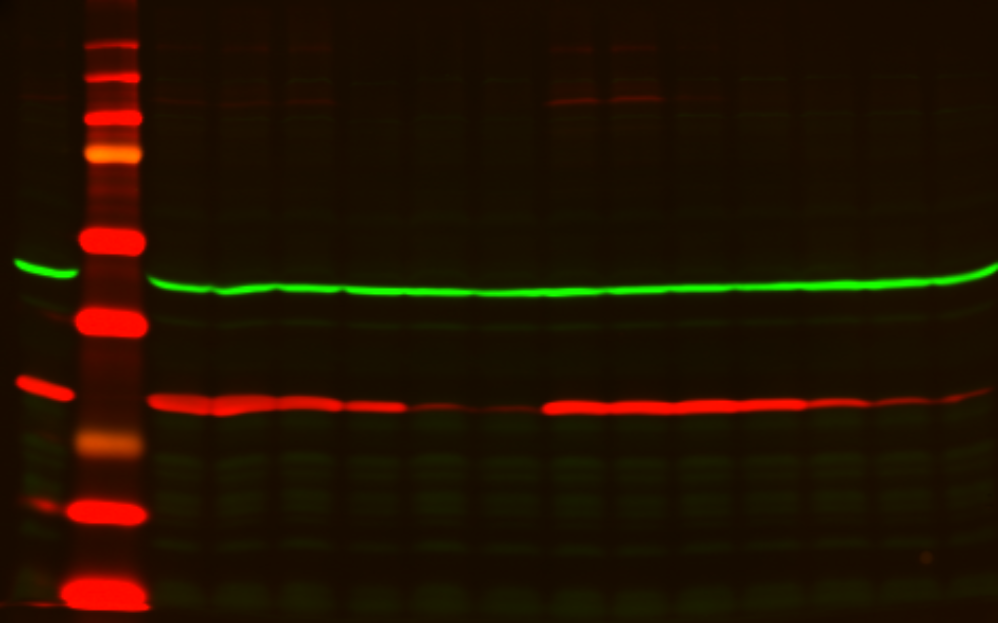

Supplement: Source data 2. [file elife-68773-data2.zip › raw gel images/Figure 8-figure supplement 3-source data 2.tif]

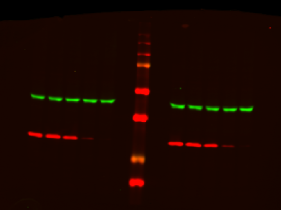

Supplement: Source data 2. [file elife-68773-data2.zip › raw gel images/Figure 8-source data 1.tif]

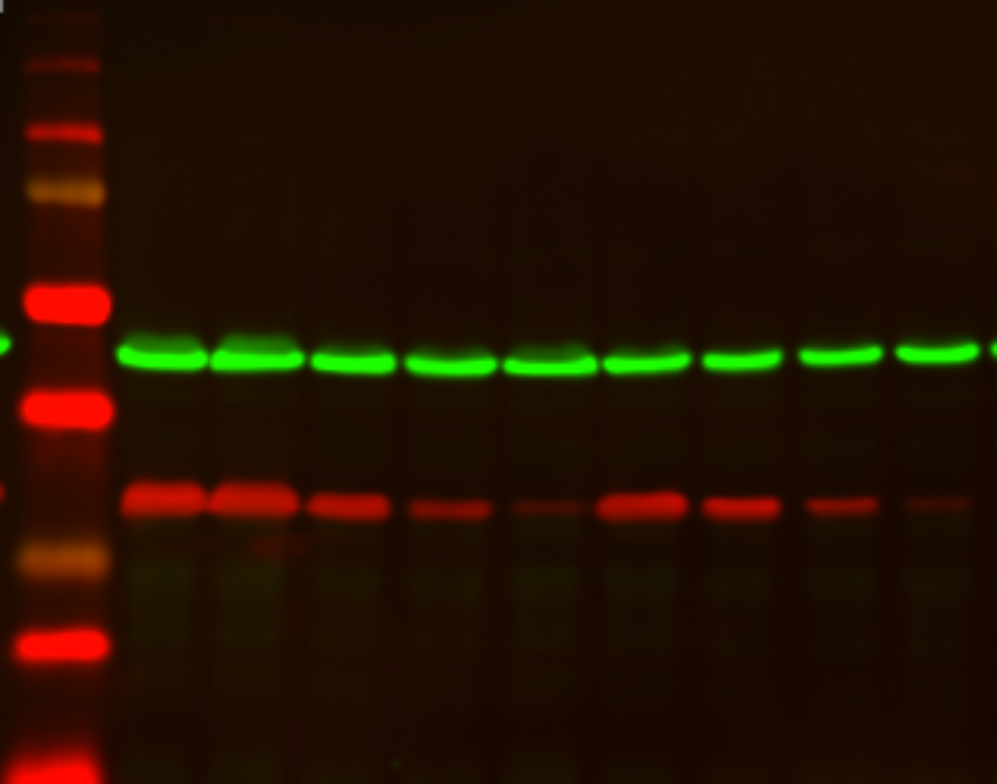

Supplement: Source data 2. [file elife-68773-data2.zip › raw gel images/Figure 6-figure supplement 1-source data 1.tif]

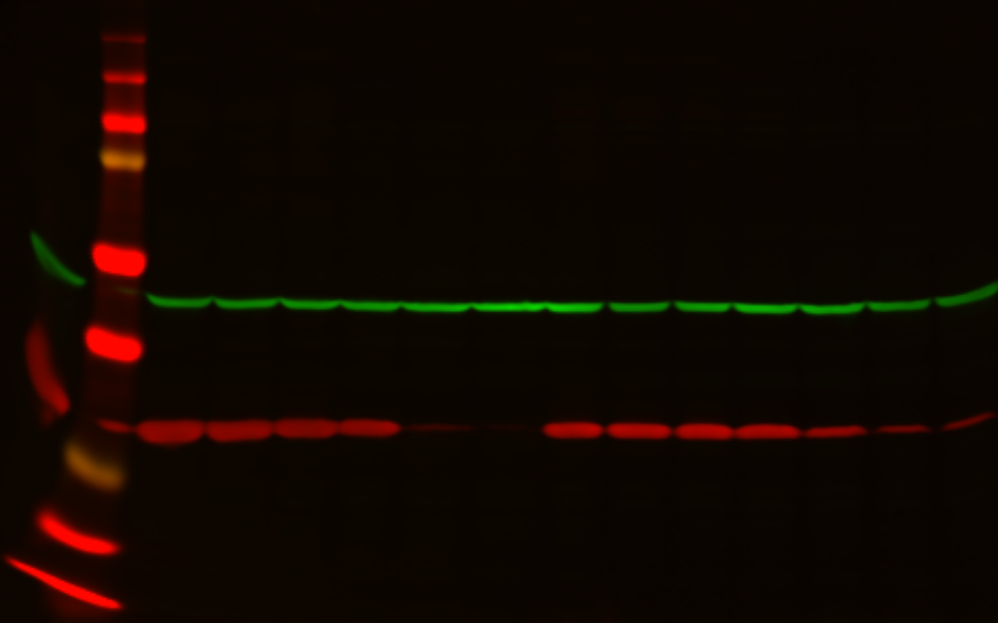

Supplement: Source data 2. [file elife-68773-data2.zip › raw gel images/Figure 8-source data 2.tif]

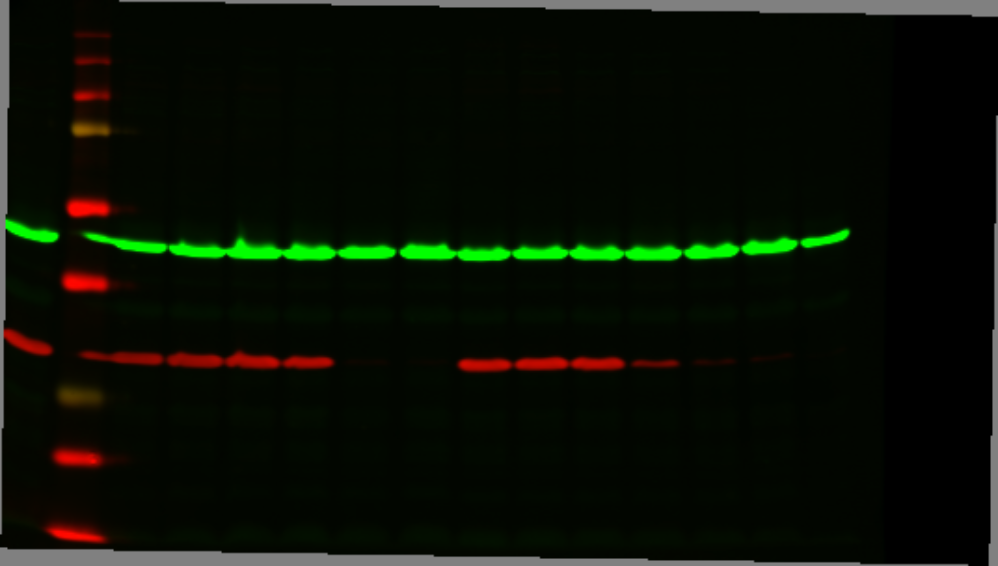

Supplement: Source data 2. [file elife-68773-data2.zip › raw gel images/Figure 8-source data 3.tif]

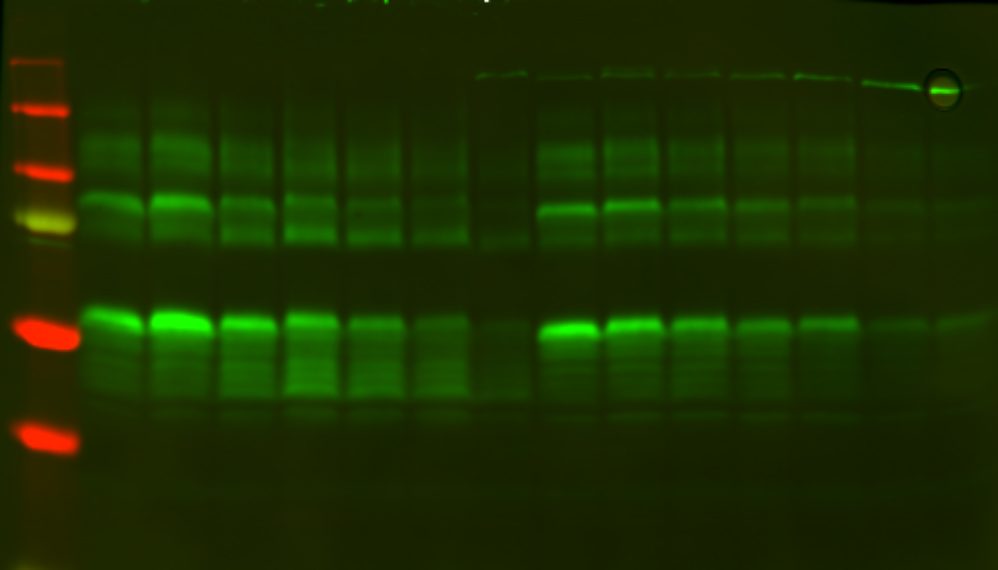

Supplement: Source data 2. [file elife-68773-data2.zip › raw gel images/Figure 6-figure supplement 1-source data 2.tif]

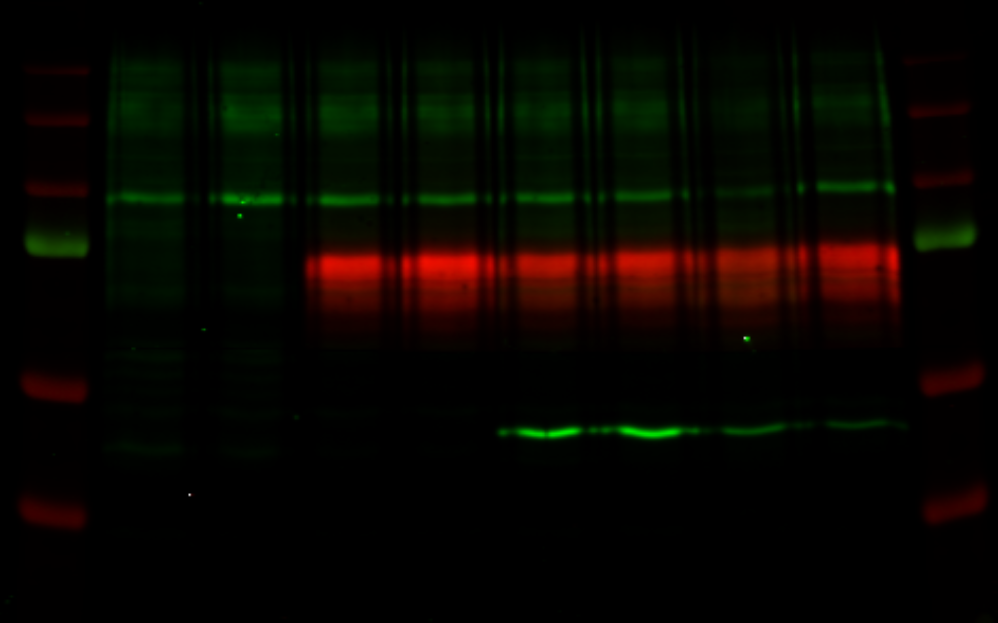

Supplement: Source data 2. [file elife-68773-data2.zip › raw gel images/Figure 9-source data 1.tif]

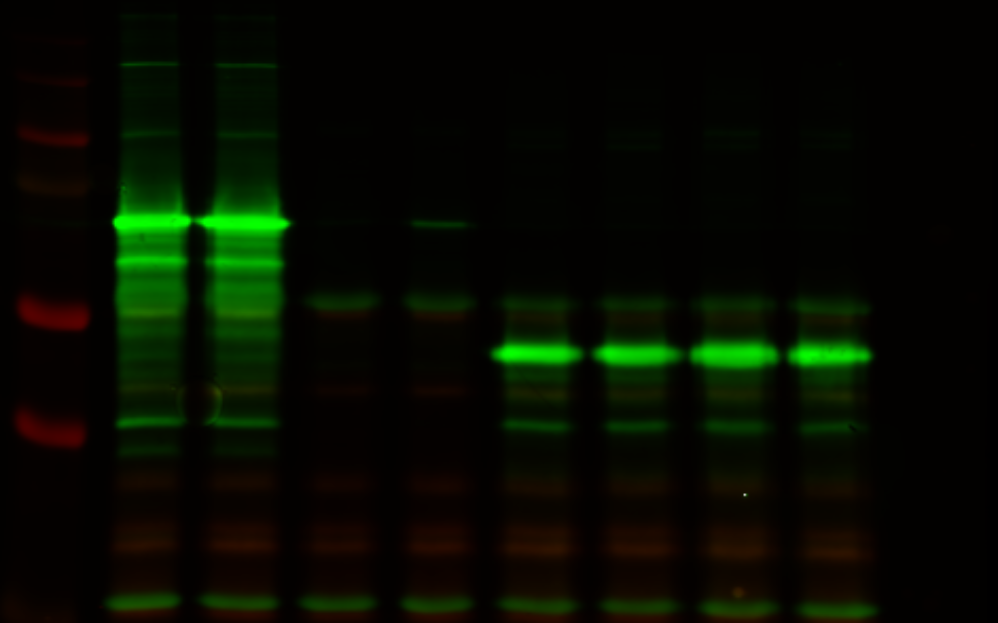

Supplement: Source data 2. [file elife-68773-data2.zip › raw gel images/Figure 9-source data 2.tif]

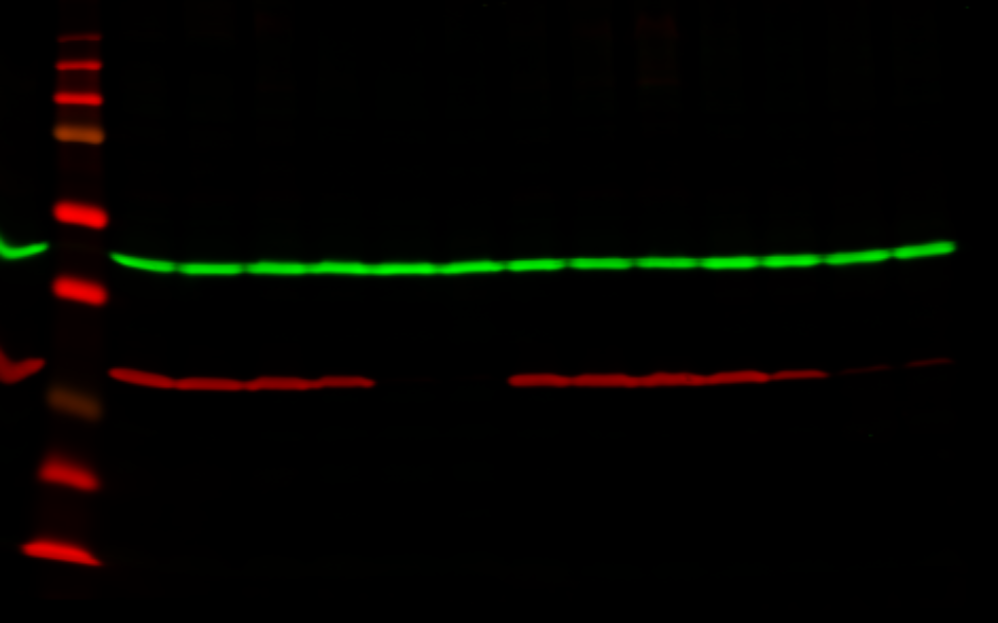

Supplement: Source data 2. [file elife-68773-data2.zip › raw gel images/Figure 8-source data 4.tif]

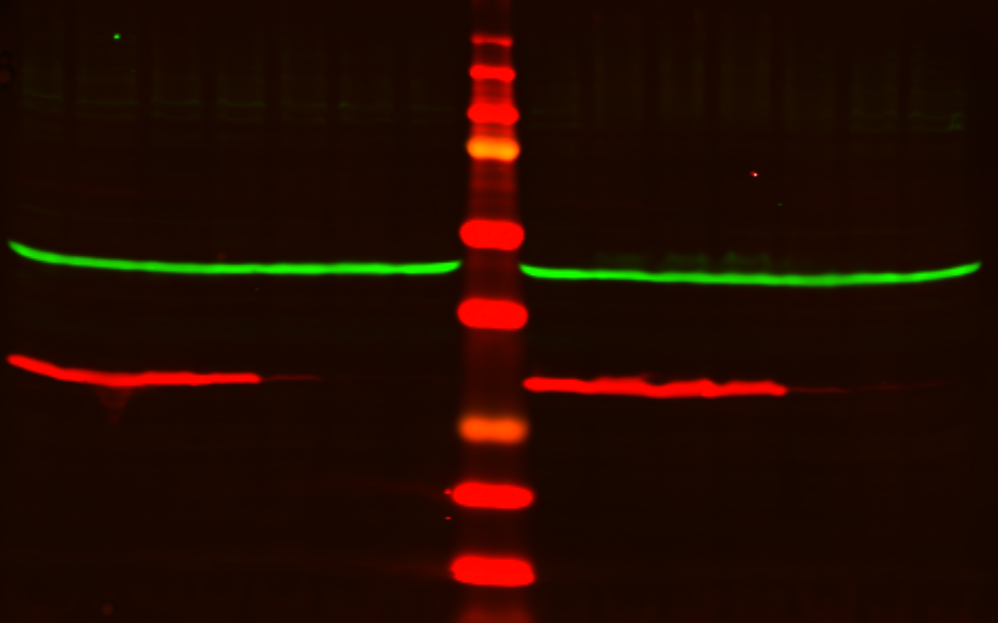

Supplement: Source data 2. [file elife-68773-data2.zip › raw gel images/Figure 8-source data 5.tif]

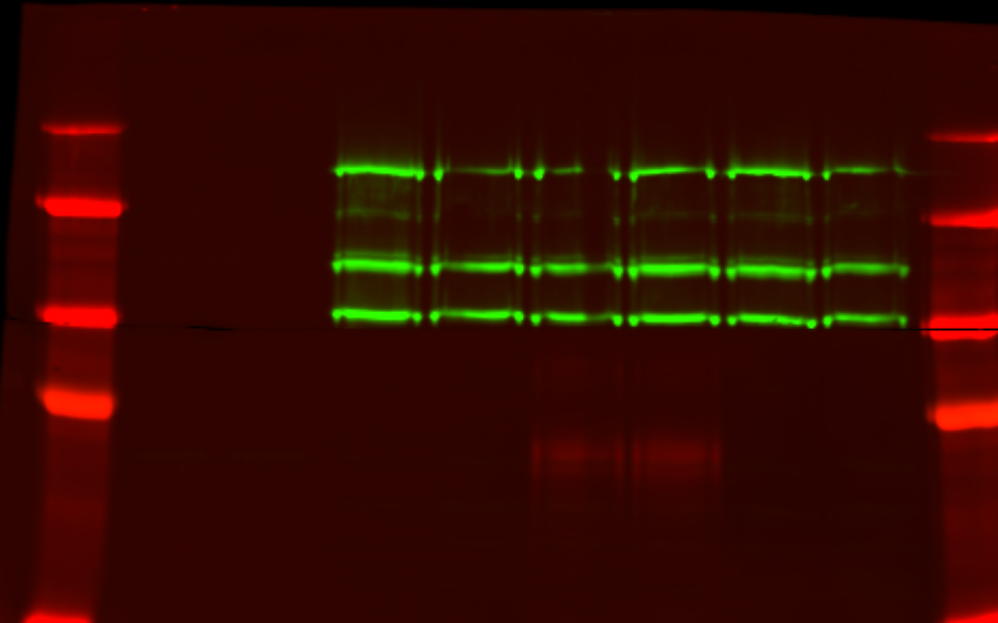

Supplement: Source data 2. [file elife-68773-data2.zip › raw gel images/Figure 3-figure supplmement 1-source data 1.tif]

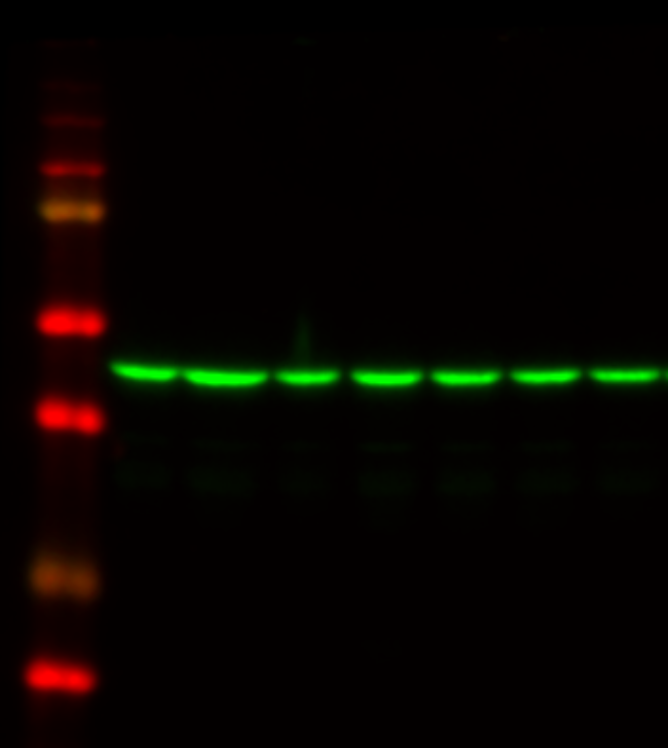

Supplement: Source data 2. [file elife-68773-data2.zip › raw gel images/Figure 3-figure supplmement 1-source data 3.tif]

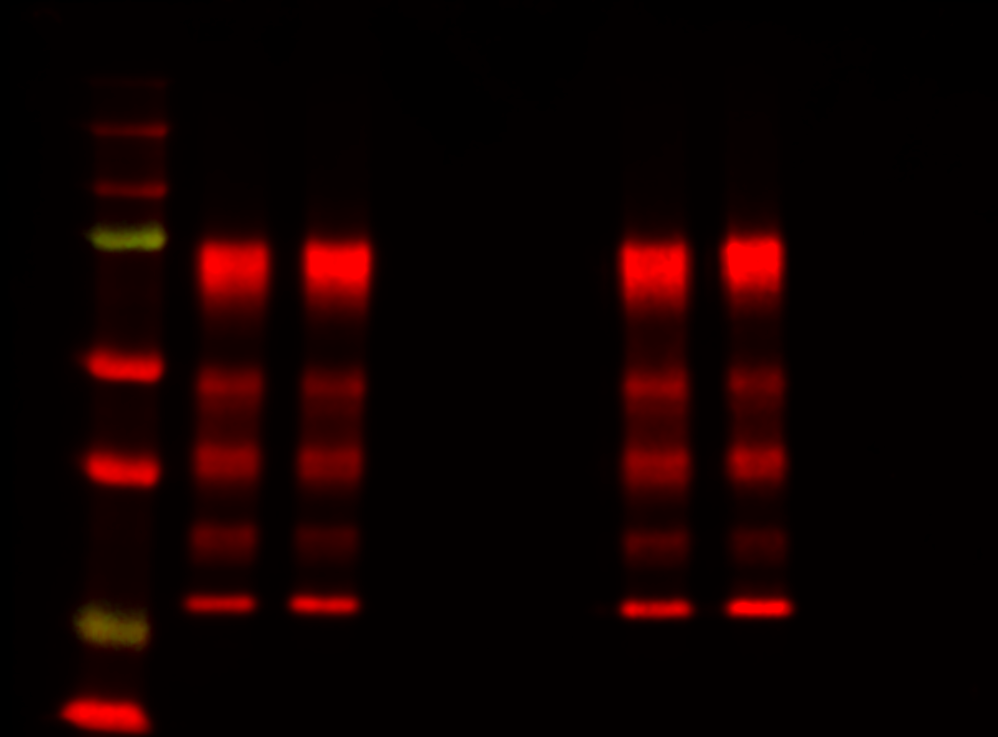

Supplement: Source data 2. [file elife-68773-data2.zip › raw gel images/Figure 3-figure supplmement 1-source data 2.tif]

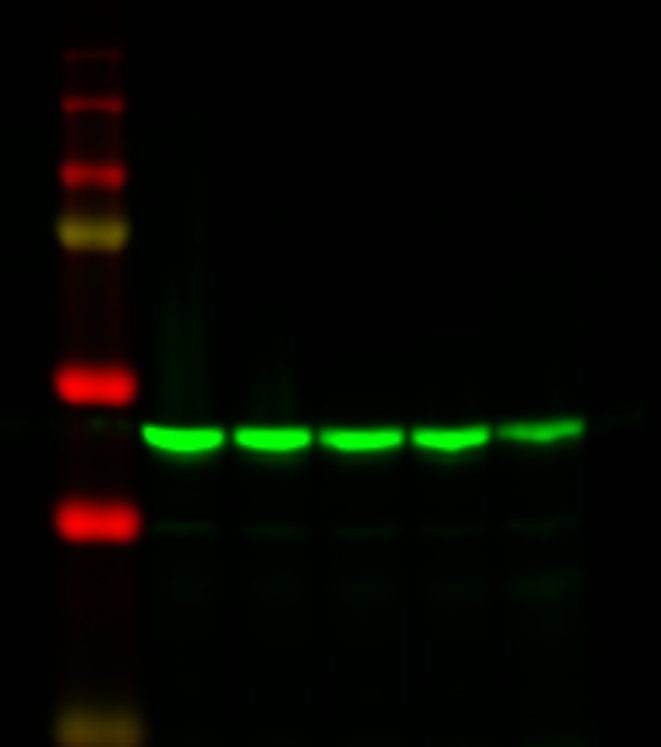

Supplement: Source data 2. [file elife-68773-data2.zip › raw gel images/Figure 3-figure supplmement 1-source data 6.tif]

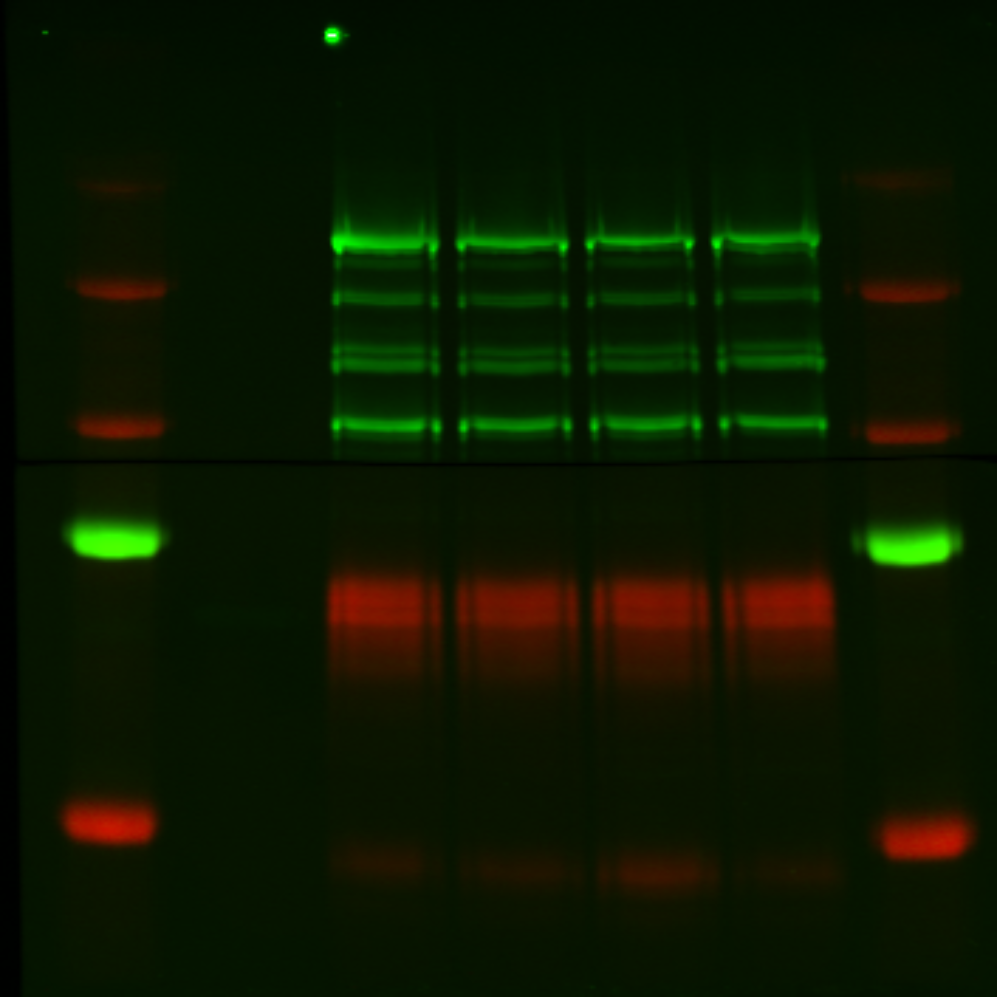

Supplement: Source data 2. [file elife-68773-data2.zip › raw gel images/Figure 3-figure supplmement 1-source data 7.tif]

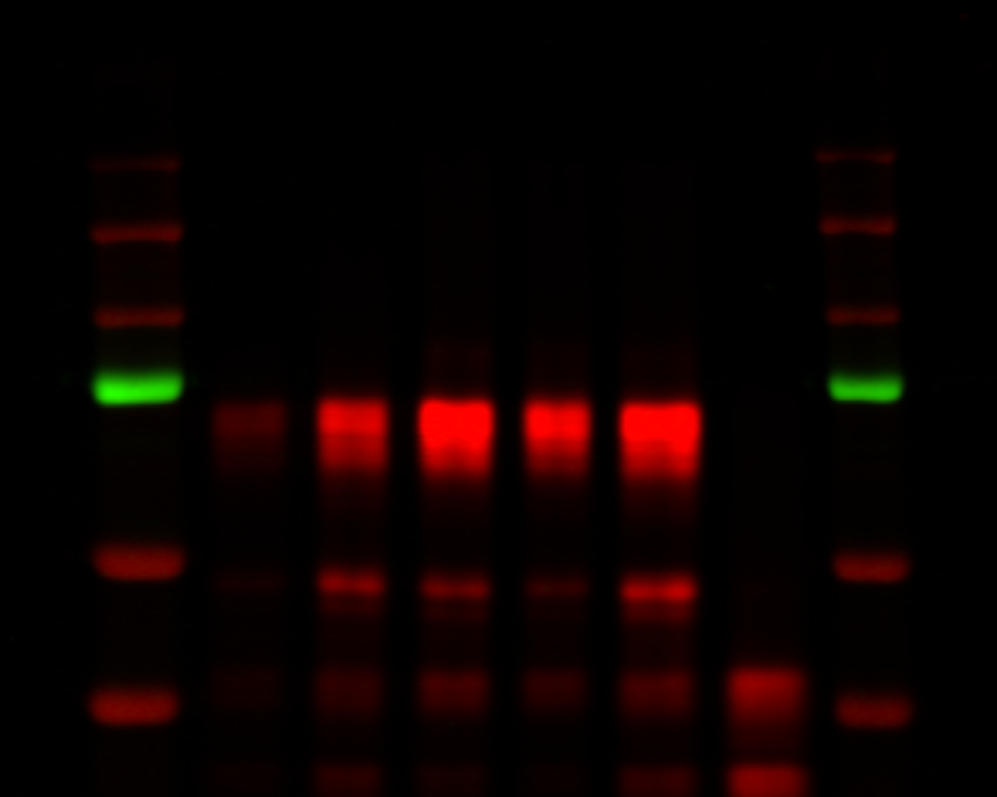

Supplement: Source data 2. [file elife-68773-data2.zip › raw gel images/Figure 3-figure supplmement 1-source data 5.tif]

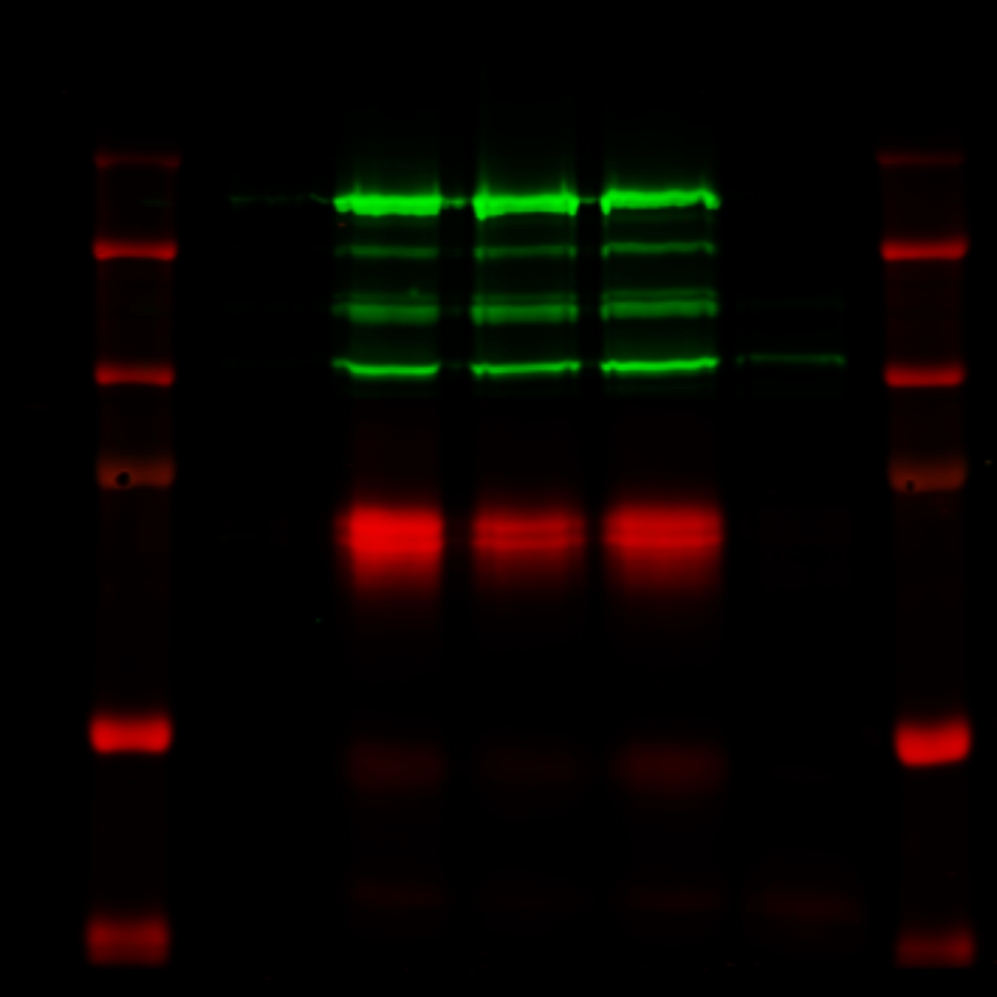

Supplement: Source data 2. [file elife-68773-data2.zip › raw gel images/Figure 3-figure supplmement 1-source data 4.tif]
